# Lineage topology, replication kinetics and cell cycle synchronization reveal regulated growth dynamics in human bone marrow stromal cell colonies

**DOI:** 10.1038/s41598-026-51809-z

**Published:** 2026-06-04

**Authors:** Alessandro Allegrezza, Riccardo Beschi, Domenico Caudo, Andrea Cavagna, Alessandro Corsi, Antonio Culla, Samantha Donsante, Giuseppe Giannicola, Irene Giardina, Giorgio Gosti, Tomás S. Grigera, Stefania Melillo, Biagio Palmisano, Leonardo Parisi, Lorena Postiglione, Mara Riminucci, Francesco Saverio Rotondi

**Affiliations:** 1 Physics Department, Sapienza University, Rome, Italy; 2 Center for Life Nano & Neuro Science, Italian Institute of Technology, Rome, Italy; 3 Institute for Complex Systems, National Research Council, Rome, Italy; 4 Istituto Nazionale di Fisica Nucleare, Sezione Roma 1, Rome, Italy; 5 Department of Molecular Medicine, Sapienza University, Rome, Italy; 6 Department of Anatomical, Histological, Forensic Medicine and Orthopedic Sciences, Sapienza University, Rome, Italy; 7 Instituto de Física de Líquidos y Sistemas Biológicos, CONICET and Universidad Nacional de La Plata, La Plata, Argentina; 8 CCT CONICET La Plata, Consejo Nacional de Investigaciones Científicas y Técnicas, La Plata, Argentina; 9 Departamento de Física, Facultad de Ciencias Exactas, Universidad Nacional de La Plata, La Plata, Argentina; 10 Present Address: Tettamanti Center, Fondazione IRCCS San Gerardo dei Tintori, Monza, Italy; 11 Present Address: Institute of Heritage Science, National Research Counci, Rome, Italy; 12 Present Address: Dipartimento di Ingegneria Chimica, dei Materiali e della Produzione Industriale, Universita degli Studi di Napoli Federico II, Napol, Italy

**Keywords:** BMSCs, Skeletal stem cells, Single-cell analysis, Regenerative medicine, Senescence, Cell proliferation, Lineage tracing, Time-lapse microscopy, Non-interacting branching process model, Cell biology, Stem cells

## Abstract

Bone marrow stromal cells (BMSC) – which include skeletal stem cells – are a promising tool in regenerative medicine. However, their heterogeneous and unpredictable *in vivo* behaviour remains a critical barrier preventing the development of standardized therapeutic approaches for skeletal tissue regeneration. Several studies have attempted to identify *in vitro* features that could correlate with the *in vivo* differentiation properties, yet the mechanisms ruling BMSC heterogeneity remain poorly understood. Here, using time-lapse imaging, we lineage-trace 32 single-cell-derived BMSC colonies through seven generations. We observe significant inter-colony and intra-colony heterogeneity in lineage topology (determined by the number of senescent or apoptotic cells) and in replicative kinetics (measured from proliferating cells only). Interestingly, topology and kinetics are strongly correlated, suggesting the existence of regulatory factors linking the non-dividing/apoptotic subpopulations with proliferating cells. Furthermore, BMSCs display a high degree of cell-cycle synchronization during early generations, indicating stage-specific regulatory mechanisms through which cells influence each other. By employing a non-interacting population growth model, we demonstrate that the observed synchronization cannot be explained by an uncorrelated branching process; instead, correlated division times among cells must be present. Our findings reveal fundamental mechanisms governing BMSC heterogeneity and growth dynamics that may inform strategies to control their regenerative potential.

## Introduction

Bone marrow stromal cells (BMSCs) can be isolated from the post-natal bone marrow and cultured *in vitro* as adherent, fibroblast-like cells. When transplanted *in vivo* at heterotopic sites, they are able to generate skeletal tissues and establish a functional bone marrow microenvironment^1^. BMSCs contain a subset of cells that are able to proliferate even in low-density cultures – i.e. in the absence of paracrine stimuli produced by neighbouring cells – to form colonies^1,2^. These clonogenic BMSCs include skeletal stem cells (SSCs) and progenitor cells normally residing in the post-natal bone marrow^3–5^. Therefore, they are the repository of the regenerative properties observed in *in vivo* assays and are critical to any potential therapeutic application of BMSCs. Colony-forming BMSCs share many biological features, including the expression of specific surface markers that can be used for prospective purification^6–8^. However, the colonies that they generate are rather heterogeneous and show considerably different *in vivo* behaviour. Indeed, colonies formed by BMSCs corresponding to genuine SSCs undergo multi-lineage differentiation and organise a bone marrow microenvironment, whereas colonies derived from BMSCs corresponding to more committed progenitor cells produce only bone^4,9^. Furthermore, some colony-forming BMSCs generate a progeny that does not perform any specific function^4^.

The heterogeneous *in vivo* behaviour of BMSC colonies is one of the most important factors preventing the development of effective therapeutic approaches for skeletal tissue regeneration. Several studies have attempted to identify morphological and/or molecular features expressed *in vitro* that correlate with the differentiation properties displayed *in vivo*^9–12^. Although specific surface markers for human SSCs have been identified^13^, *ex-vivo* expansion is a required step to achieve an appropriate cell number for clinical applications; hence, predictive parameters of BMSC colony behavior are needed to assess their *in vivo* potency^14^. Therefore, new methods to sort BMSC colonies with similar potency need to be explored. Lineage studies are a promising approach in this respect. By tracing the proliferation of colonies derived from different initial cells, we can try to identify the nature of the variability between initiator cells leading to the subsequent *inter-colony* heterogeneity we wish to control. It is of particular interest whether the variability among initiator cells belonging to the same donor and expanded in the same conditions is any different from the variability across different experiments and different donors. Moreover, different parts of the same colony may develop differently and it is important to understand whether these *intra-colony* heterogeneities are strong enough to wash out any colony-level correlations, or whether these fluctuations are generated and structured in a way that keeps the collective identity of the clone.

Time-lapse microscopy has been used to reconstruct the lineage of both embryonal and adult cell colonies^15–20^. In the case of BMSCs^21–24^, the technical challenges are considerable, so that lineage studies have followed proliferation only for the first few generations (typically five). All studies discovered great heterogeneities and some proved that colonies may have significant variability in their differentiating potential^21,23^. An important aspect to keep in mind in this respect is that, even at low plating densities, BMSCs tend to cluster, so apparently isolated colonies may actually result from merged clones^8^; undetected merging dangerously confounds inter-colony and intra-colony heterogeneity, while to draw sharp conclusions about BMSC heterogeneity, and its potential correlations, it is critical to thoroughly confirm that colonies undergoing analysis indeed originated from single cells^8^. Therefore, the study of clonal colonies should be performed only through lineage tracing of isolated, single-cell-derived colonies. This approach is, however, very time-consuming; to date no studies have analyzed more than 10 single-cell-derived colonies, which is borderline for statistical analysis.

Here, we conduct a study of human BMSC proliferation *in vitro* through time-lapse microscopy of unprecedented extension and depth, reconstructing the full lineage of 32 single-cell-derived clonal colonies, traced up to the seventh generation. We characterize colonies according to two major traits: the topology of the lineage – namely how many cells reach a specific generation and how many stop proliferating – and the kinetics of the colony – expressed by the statistics of the division times. We show that, although both traits are strongly heterogeneous, fluctuations in topology and kinetics are not independent from each other, unveiling a strong correlation that connects the propensity of the colony to produce senescent and/or apoptotic cells to the proliferation properties of dividing cells. Moreover, we find that the variability observed among the colonies within each cell-culture experiment (i.e. same donor, age and passage) is as large as the overall heterogeneity across all donors, age and passages. Finally, we observe a remarkable degree of synchronization of the divisions of cells belonging to the same generation. We try to characterize this synchronization through a branching process model for a population of non-interacting individuals, but we observe a significant mismatch between theoretical model and experimental data. This fact indicates that cell cycle in BMSC colonies is much more synchronized than it would be expected from a non-interacting model, therefore demonstrating that some level of kin correlations between the division times of cells at the same generation (sister-sister, cousin-cousin, etc) must exist.

## Methods

Detailed methods are given in the Supporting Information (SI) for better manuscript readability; here we present a brief summary of the most salient traits of the experiment.

### Cell culture

Data were collected during the course of 11 experiments, from January 2023 to June 2025. Primary human bone marrow stromal cells (BMSCs) were isolated from humerus and radius bone samples obtained from 6 healthy donors undergoing elbow orthopaedic surgery. All procedures were approved by the local ethics committee (Ethics Committee Lazio, Rif. 5313 Prot. 387/19) and performed in accordance with the Declaration of Helsinki and relevant guidelines and regulations, with informed consent from all donors. BMSCs were separated from hematopoietic cells based on their adherence to plastic culture dish and cultured in *α*-MEM medium supplemented with 1% Penicillin/Streptomicin 1% L-glutamine and 20% Fetal bovine serum^25^. We did not perform “Mesenchymal Stem Cell (MSC)” characterization, as the establishment of multi-colony cultures by plastic adherence produce highly pure BMSC cultures^26^. Cells for time-lapse study were plated at passage P0, P1, P2, or P3 depending on the experiment (see Supplementary Table S1).

### Single-cell-derived clones

To draw conclusions about intra- vs inter-colony heterogeneity, it is imperative to be certain that colonies in the dataset indeed originated from single cells; hence, an experimental protocol that enables accurate identification of single-cell-derived colonies is a crucial component of well-controlled BMSC studies^24^. In order to ensure that only single-cell-derived colonies were included in our study, at the beginning of the experiment we only select single cells that are completely isolated, so that we are sure of the true clonal nature of each one of our colonies. To this aim, cells were seeded in 35 mm culture dishes at very low density (10–20 cells/dish), to increase the probability to form colonies derived from single spatially isolated cells. Moreover, colonies that got in contact with each other, or that had cells falling out of the field of view, were discarded.

### Experiment duration

In contrast to most time-lapse experiments, we do not fix the maximum time duration of the experiment, but rather the maximum generation *k*, keeping the experiment going until all proliferating cells in a colony reached *k=7*. In this way there is no need to correct for the bias in the statistics of the division times and in the reconstruction of the lineage tree (the main features we wish to investigate) that is introduced if the total observation time is fixed^27^. Notice that tracking 32 clones up to generation seven constitutes an unprecedented lineage depth study. Tracking cells beyond generation *k=7* becomes extremely time-expensive and problematic for the image-analysis (see Supplementary Methods for more details about cell tracking); for only three colonies—in order to test the predictive power of the branching process model—we track proliferation up to generation *k=8*.

### Time-lapse imaging and tracking

The growth of the colonies was studied through time-lapse, using a Nikon Inverted Eclipse Ti2-U phase contrast microscope, equipped with a Nikon DS-Qi2 14 bit monochrome camera, with a resolution of 16.25 megapixels, recording high resolution images at 20*×* every 15–20 minutes, depending on the experiment (see Supplementary Table S1). To work out the lineage of a colony it is necessary to determine the relationships between mother and daughter cells, and to work out precisely all division times. To ensure unequivocal identification of cells and mitosis, the tracking of individual cells was performed manually via a custom semi-automatic software. No unsupervised algorithm has been used.

**Fig. 1:**
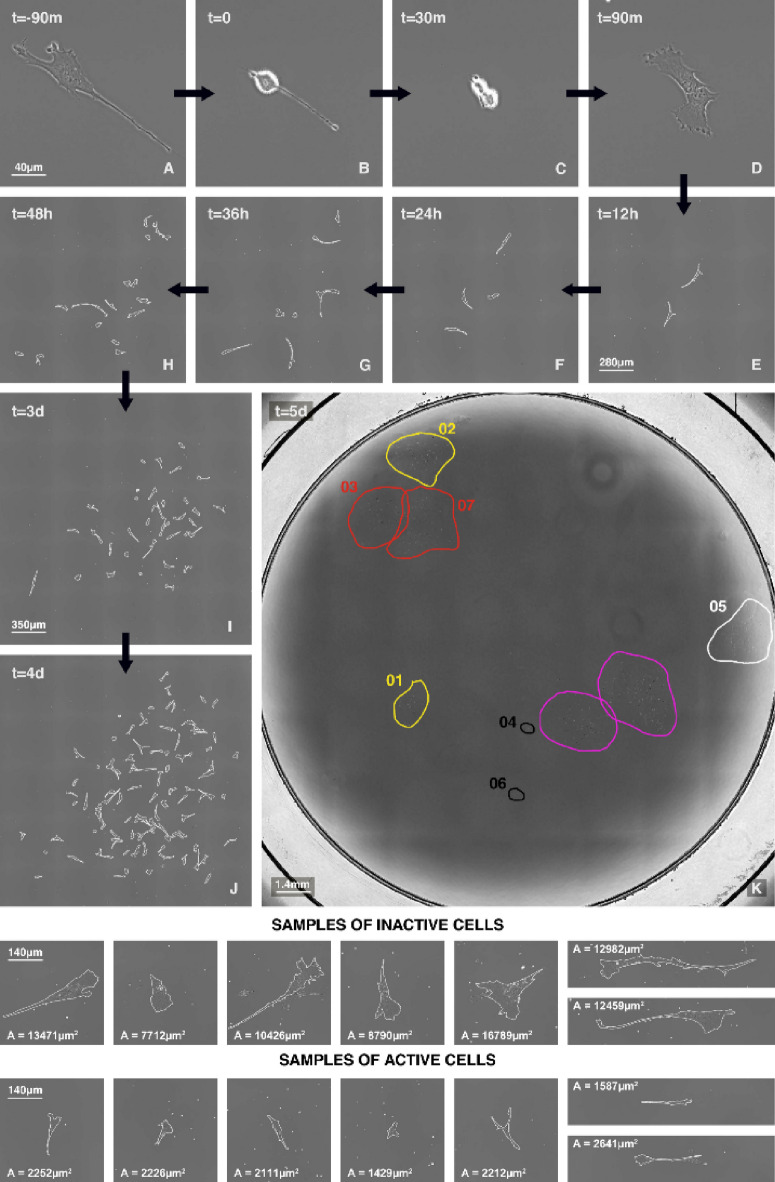
Time-lapse of a BMSC colony. (**A**–**K**) Images follow the evolution in time of colony 20241112_05 from its single originating cell. Panels (**A**–**J**) are different scale crops of images acquired at magnification *20×*. (**A**) The originating cell 90 minutes before its mitosis. (**B**) During the Mitotic Rounding Phase (MRP), cells assume a bright and almost spherical shape. We use the MRP as a marker for mitosis. The MRP of the originating cell marks the temporal beginning of the colony development defining the time origin (*t=0*) for the colony. (**C**) Half an hour after the beginning of MRP, the two daughter cells still have a spherical shape. (**D**) At *t=90* minutes, the two daughters are distinguishable by their well-separated nuclei. (**E**–**H**) Within the first 48 hours, the colony expands to the fourth generation. (**I**,**J**) During the third and the fourth days this colony expands to the fifth and sixth generation. (**K**) Overview at magnification *4×* of the entire dish at day five (*8× 8* photo tessellation). The white contour encloses the colony described in panels (**A**–**J**). Yellow contours indicate isolated colonies that we still follow on day five; red contours indicate infiltrated colonies (colonies that got in contact with each other), which we stop recording. Purple denotes colonies derived from cells that we did not select at the beginning of the experiment because of their close proximity. Finally, black contours correspond to cells that were selected but never formed colonies. Bottom panels. Examples of $\textrm{G}_0$ and active cells at the same magnification. $\textrm{G}_0$ cells have a different –flat– morphology, and are larger than the spindle-shaped active cells.

## Results

### Description of the clones

Out of the 10–20 cells that are present in the dish at the beginning of each experiment, between 65% and 70% of them start proliferating and form a colony; however, not all these colonies were included in the analysis, either because they come from cells that were not well-isolated at the beginning, or because the colonies infiltrate each other; in other cases, a clone is well-isolated, but it grows too close to the border of the plate, so that some of its cells crawl on top of the plate’s border and go out of focus, making the data unusable. On average, between 2 and 5 clones per experiment were included in the analysis. Following these lines, we obtained in total 32 single-cell-derived BMSC clonal colonies. Fig. 1 shows representative time-lapse images following the growth of one of the colonies, from the single cell scale up to the entire dish; the reader can also refer to Supplementary Video S1 for the full time-lapse evolution of one colony and to Supplementary Fig. S1 for more images of the colonies at generation seven.

### Active and inactive cells

The maximum number of cells at the seventh generation is $2^7=128$, and indeed a handful of our lineages reach this condition; this happens when all cells in the colony remain *active* up to generation seven; here, we define as active all cells that undergo mitosis and hence have a well-defined division time. However, not all cells continue to proliferate, hence not all colonies at *k=7* have 128 cells; some cells stop dividing, yet remain alive, thus entering the $\textrm{G}_0$ phase^28–30^. We classify a cell as $\textrm{G}_0$ if it does not divide within *84 *hours (*3.5 *days) from its birth; this threshold is much larger than the mean division time of BMSCs, which is $20 \pm 4\,$hours. In the Supplementary Notes section we demonstrate that this criterion is robust, so that our results do not depend on the specific threshold of 84 hours. This value of the threshold is larger than what previously adopted in studies on BMSCs to classify non-dividing cells^24^, and it is consistent with the experimental and tracking constraints due to cell crowding at the largest considered generation. $\textrm{G}_0$ cells can be quiescent (able to reverse to a proliferating state) or irreversibly non-proliferating (senescent or differentiated)^29,31^. A previous study of BMSC colonies^22^ established through the *β*-galactosidase assay that most $\textrm{G}_0$ cells were senescent —although that study used passage P5, higher than our P0–P3. Moreover, several studies^22,32,33^ established a clear correlation between senescent state and morphology: senescent cells typically have a larger area and a flat morphology, very different from the spindle-like shape of proliferating cells, and present a reduced motility; this is also the case for our $\textrm{G}_0$ cells (see Fig. 1, bottom and Supplementary Fig. S2). Nevertheless, since we did not run the *β*-galactosidase assay, we prefer to use the less committed label $\textrm{G}_0$ for cells that stop dividing.

In very few cases, cells commit apoptosis and die (apoptosis is very clear in the imaging, as the cell bursts and detaches from the plate). Despite the obvious biological difference between $\textrm{G}_0$ and dead cells, they have the same effect on lineage topology, namely to interrupt a branch. It is therefore convenient to use a term that encompasses both $\textrm{G}_0$ and dead cells: we call them *inactive.* In this way, the total population of a tree is given by the union of its active and inactive cells, while the inactive sub-population is the union of $\textrm{G}_0$ and dead cells. In our case, most of the inactive cells are $\textrm{G}_0$: we recorded only 17 dead cells in our whole dataset, which is just 5% of all 327 inactive cells (see Supplementary Table S1).

### The lineage trees

**Fig. 2:**
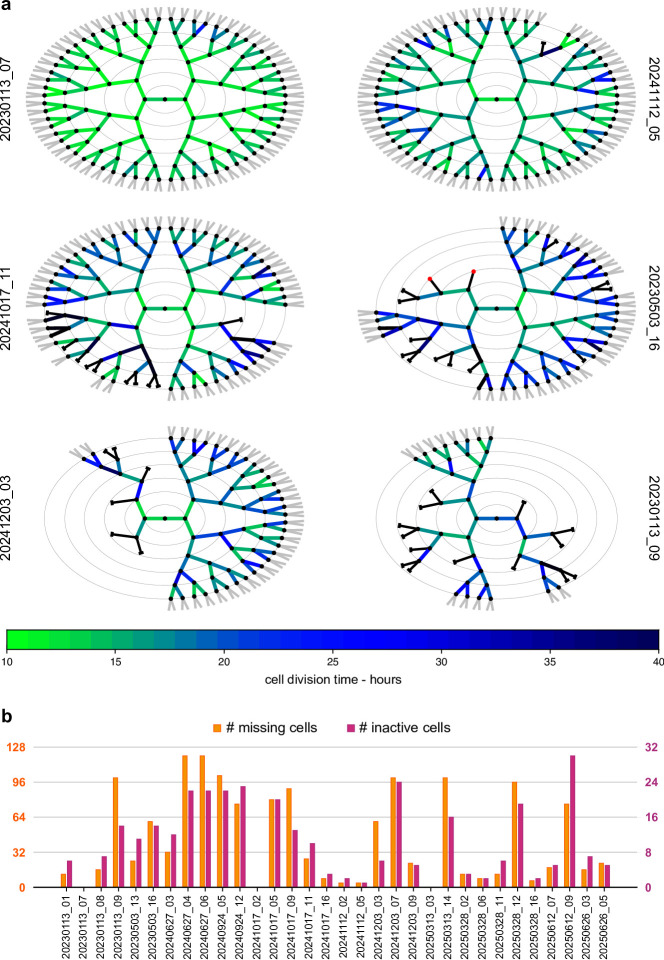
Topology — Lineage trees. (**a**) Abstract representation of the lineages of six BMSC colonies in the dataset. Each node (black dots) represents a mitosis and each link between two nodes represents a cell; the central node is the mitosis of the originating cell (the originating cell is not represented in the tree). All links have the same length, while their color represents the division time of the corresponding cell: light-green for short division times, dark-blue for long times (the mean division time across the whole dataset is about 20 hours). The thin elliptical lines separate different generations. All colonies have been followed up to the seventh generation, which means that the last divisions we observe are those between generation *k = 6* and *k = 7*; hence, we do not measure the division times of the *k = 7* cells, which are therefore colored grey. In some colonies all cells divide up to the seventh generation, giving rise to $2^7=128$ cells (20230113_07, top left), but in most colonies some inactive cells ($\textrm{G}_0$ or dead) emerge. We put a short black cap at the end of the link to represent a $\textrm{G}_0$ cell, or a red dot for a dead cell. Inactive cells do not have a division time and we color them in black. (**b**) To show the significant inter-colony heterogeneity of the topology, we report for each lineage in the dataset the number of inactive cells and the number of missing cells at the seventh generation. Both quantities have fluctuations that are almost as large as the mean.

We adopt an abstract radial representation of the lineage trees (Fig. 2a), similar to that of Refs.^34,35^: each node represents a mitosis and each link represents a cell. Unlike in more standard representations^22,24^, all links have the same length (so that every lineage occupies the same area), and the division time (i.e. the cell cycle duration) of the cell is represented by the link’s color (light green for a fast cell cycle and dark blue for a slow cycle). Since we do not know the time of birth for the first cell, there is no link to represent it; the mitosis of this first cell (the root of the tree) defines the origin of time for the colony (*t=0* in Fig. 1). Mitosis of cells belonging to the same generation lie on the same ellipse, while cells of the same generation are represented by segments lying in the region between two consecutive ellipses. Because we trace cells up to generation *k=7*, the last divisions we observe are those between *k=6* and *k=7*; accordingly, *k=7* cells are colored grey because they lack a recorded division time.

An inactive cell has no mitosis or division time, hence we conventionally paint it black and put a black cap at the end of the link for $\textrm{G}_0$ cells, or a red circle for dead cells (Fig. 2a). We stress that this abstract representation contains no information about physical positions and mutual distances between cells; although there is some correlation between distance on the tree and physical distance, it is not very stringent (see^36^ for an in-depth analysis of this point).

The bare backbone of a lineage – how cells are connected to each other, independently of the color of the links (division times) – is what we call the *topology* of the tree. This is exclusively determined by the location of the inactive cells. The overall structure of the colony is given by the distribution of links plus the division times (i.e. the links’ color, or length if one uses the standard representation^24^). Thus different division times will produce different structures for the same topology. In other words, the structure is the combination of topology (how inactive cells are distributed) and kinetics (how fast the active cells divide). Since we aim here at disentangling the roles of these two elements, we have chosen to use the words ‘topology’ and ‘kinetics’, rather than ‘structure’.

### Heterogeneity of topology

In Fig. 2a we show a subset of our lineage trees: a qualitative inspection reveals great diversity in the trees’ topologies, confirming, at the lineage level, what previous studies have already established for other parameters^8–10,22–24^, namely that BMSC populations display a great *inter-colony* heterogeneity. We find, on one hand, perfectly whole lineages, with all branches reaching the last recorded generation (3 out of 32 colonies in our dataset, as 20230113_07 in Fig. 2a), while on the other hand there are cases of very broken trees, with many branches interrupted by inactive cells (e.g. 20230113_09 in Fig. 2a). A first way to quantify the heterogeneity of the colonies is to measure for each one of them the number of inactive cells, $N_\textrm{inactive}$; this number has fluctuations almost as large as the mean, $N_\textrm{inactive}= 10.38 \pm 8.42$ (mean ± sd), i.e. relative fluctuation of *81.4%* (Fig. 2b).

Given a certain number of inactive cells, their effect on the topology depends on their placement among generations: a $\textrm{G}_0$ emerging at the second generation cuts a far larger number of cells at generation *k=7* than a $\textrm{G}_0$ at the sixth generation, which only cuts two. We can quantify this effect by counting the number of *k=7* missing cells, $N_\textrm{missing}$, compared to a complete tree. Naively one may expect that $N_\textrm{inactive}$ and $N_\textrm{missing}$ are simply proportional to each other, but this is not true: although the two quantities are broadly related (in particular they are both zero in a complete tree), the value of $N_\textrm{missing}$ given $N_\textrm{inactive}$ depends on the position of the inactive cells; hence, there is no function linking these two parameters. We show in Fig. 2b that the number of missing cells varies from colony to colony even more strongly than the number of inactive cells, $N_\textrm{missing}= 44.4 \pm 40.77$, a relative fluctuation of *92%*. Hence, topology is very heterogeneous not only because of the number of inactive cells, but also because of the variability in the generation at which they emerge.

Fig. 2a also shows significant *intra-colony* heterogeneity: if we look at a single lineage, we find branches with many interruptions and other branches that instead proliferate in a balanced manner. We examine potential mechanisms underlying heterogeneity among different branches of a single tree – and their potential hereditary origin – in a separate study^36^. Here, we concern ourselves with another aspect of the lineages: whole trees have overall a lighter color than broken ones, i.e. the active sub-population is on average faster the less broken is the lineage (Fig. 2a). This potential correlation is non-trivial, as the division time is of course a trait of the active cells, while the broken topology is caused uniquely by the inactive cells, these subsets being complementary. To make this observation quantitative, we turn our attention to the analysis of the division times of active cells.

### Heterogeneity of kinetics

**Fig. 3:**
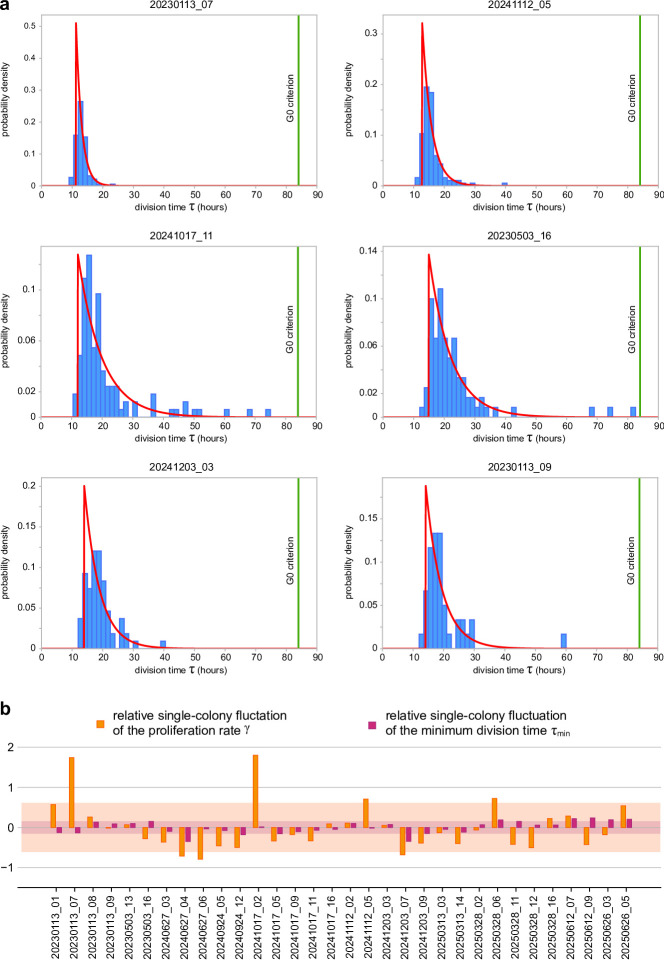
Kinetics — Statistics of the division times. (**a**) We report the probability density of the cells’ division times, *τ*, for the same six colonies as in Fig. 2a. The vertical green line in each histogram indicates the 84 hours threshold that we use to define $\textrm{G}_0$ cells. In all colonies there is a threshold below which we find no division times. For this reason, a reasonably good fit of the data (full lines) is given by the gap-exponential form (see text), which is characterized by a minimum division time $\tau _\textrm{min}$ and by a rate *γ* of decay of the exponential part of the function (red line). (**b**) We report the relative single-colony fluctuations of the rate *γ* and of the minimum time $\tau _\textrm{min}$ defined as $(x - \bar{x})/\bar{x}$, where *x* represents the observable (either $\tau _\textrm{min}$ or *γ*) and the average $\bar{x}$ is computed over all the colonies. As in the topology, a significant heterogeneity is detected also in the kinetics, in particular in the fluctuations of the rate *γ*, while the minimum division time, $\tau _\textrm{min}$, fluctuates much less. The orange and purple shading represent the standard deviation of the single-colony fluctuations of *γ* and $\tau _{min}$ respectively over all the colonies.

In Fig. 3a we show the normalized histogram (probability distribution) of the division times *τ* of active cells for the same colonies that appear in Fig. 2a (see Supplementary Fig. S3 for the complete dataset); the division time is defined as the time difference between a cell’s mitosis and its mother mitosis. The green vertical line indicates the threshold used to classify cells as $\textrm{G}_0$ ($t_c=84\,$hours); clearly $t_c$ is quite far from the whole bulk of the distribution, hence the separation of the population into active and inactive sub-populations is a qualitative one (see Supplementary Notes for a careful assessment of this point).

Models of cell proliferation in which division events occur at a constant rate *γ*^37,38^, predict that the probability distribution of the division times is a simple exponential, $P(\tau ) = \gamma \, e^{-\gamma \tau }$ (see Supplemental Equations for the detailed derivation of this general result). Although exponential decay is indeed consistent with the large *τ* tails of the experimental distributions, a pure exponential probability has its peak at *τ =0*, while all our experimental histograms show a clear gap for small division times. This feature has been observed in several systems, inspiring a different class of models^39–42^ based on the assumption that no divisions will happen below a minimum value, $\tau _\text {min}$, and that only after this time cells divide with a constant rate *γ*. This assumption leads to a simple modification of the pure exponential distribution of the division time *τ*, namely a gap-exponential,

1 $$\begin{aligned} P(\tau ) = \gamma \; \theta (\tau -\tau _\textrm{min}) \; e^{-\gamma (\tau -\tau _\textrm{min})} . \end{aligned}$$

According to equation (1), the division time *τ* cannot be smaller than $\tau _\textrm{min}$ (the Heaviside function is *θ (x)=1* if *x\ge 0* and *θ (x)=0* if *x<0*), and beyond $\tau _\textrm{min}$ a pure exponential decay with rate *γ* sets in. We fit equation (1) to the division time data, finding the values of the proliferation rate *γ* and minimum division time $\tau _\textrm{min}$ for each colony. Even though other forms could be proposed^40,43,44^, we see from Fig. 3a that distribution (1) fits reasonably well the data in most of the colonies. Notice that the mean division time relative to distribution (1) is given by (see Supplemental Equations),

2 $$\begin{aligned} \left\langle \tau \right\rangle = \tau _\textrm{min}+ \frac{1}{\gamma } \ , \end{aligned}$$

where we use the notation $\left\langle \tau \right\rangle$ to distinguish it from the experimental mean division time, $\overline{\tau }$, obtained by performing the average over all experimental division times.

**Fig. 4:**
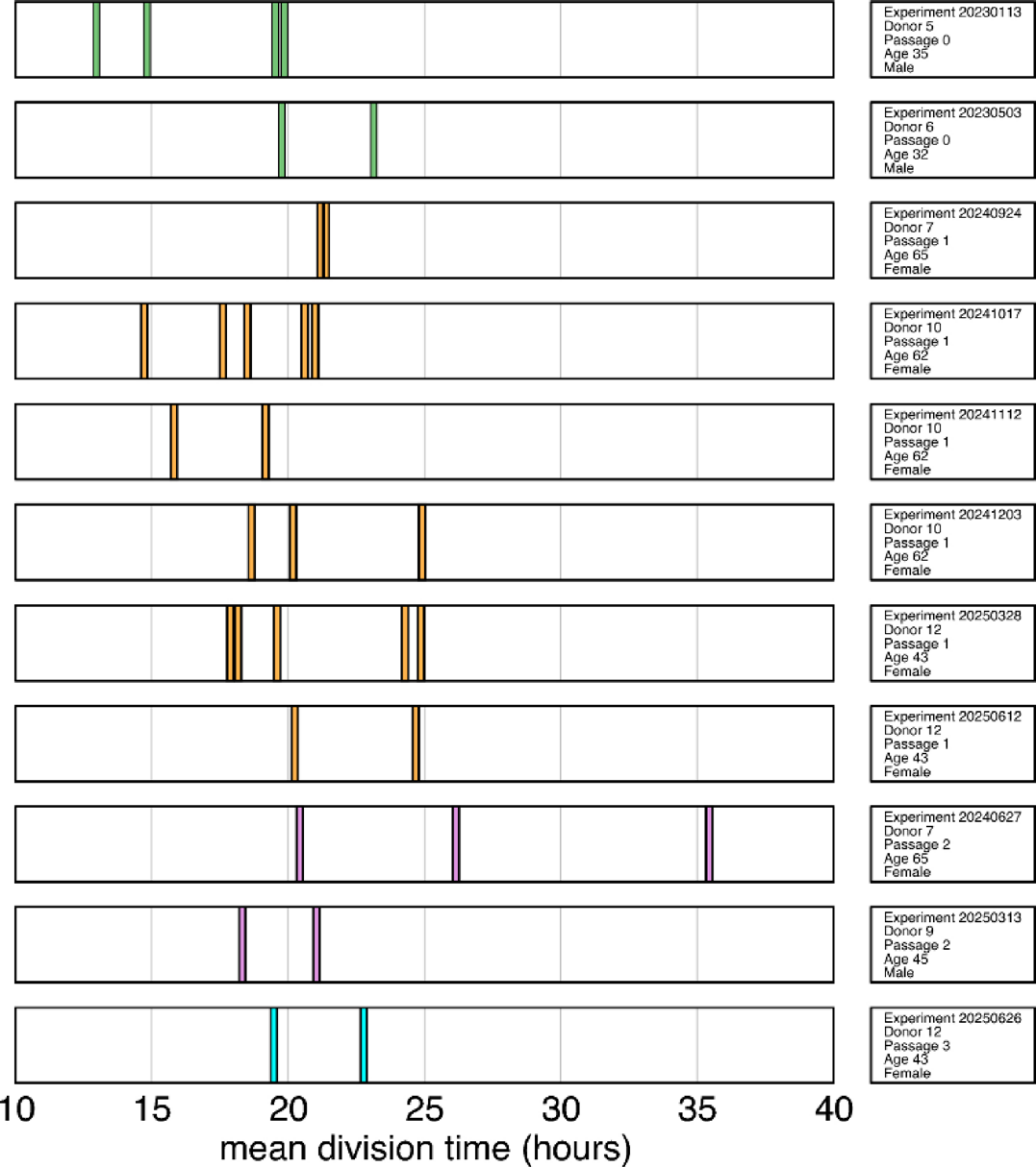
Variability with experiment, donor, age, sex, and passage. We report here the mean division time for each colony in the dataset, ordered according to experiment, donor, age, sex, and the passage of the cell culture. Each vertical bar corresponds to a different colony; colour represents passage: P0-green, P1-orange, P2-pink, P3-cyan. Fluctuations within the same experiment are of the same order as the variability across the whole dataset. The only (very weak) correlation in the data is between division time and passage (Spearman correlation *ρ =0.30*, *P*-value*= 0.046*). We notice that the only colony that seems an outlier in this plot is 20240627_06 (largest division time of experiment 20240627); interestingly, this is also the colony most separated from the bulk of points in the topology vs kinetics plot (see Fig. 5).

The relative fluctuations of $\tau _\textrm{min}$ and *γ* are given in Fig. 3b, whence we notice something interesting: while the proliferation rate *γ* strongly fluctuates from colony to colony ($\gamma = 0.19 \pm 0.12\,$ $\textrm{h}^{-1}$; relative fluctuation of *63%*), the minimal division time, $\tau _\textrm{min}$, is significantly more stable ($\tau _\textrm{min}= 13 \pm 2\,$h; relative fluctuation of *15%*). This suggests that the division time is made of two different contributions: a quasi-deterministic part, given by $\tau _\textrm{min}$, which is roughly the same for all cells, and a stochastic part, which fluctuates from cell to cell and whose corresponding rate *γ* fluctuates from colony to colony. This result is consistent with the fact that cell cycle stages S, $\textrm{G}_2$ and M are quite stable in duration, while $\textrm{G}_1$ fluctuates much more^28,39^, both in embryonal and somatic stem cells^30,45^; such variability is associated to proliferation regulation and cell fate determination^30,46,47^. We note that $\tau _\textrm{min}$ takes approximately 63% of BMSC cycle duration, while *1/γ* takes 37% of it; interestingly, in rapidly replicating human cells, the combined duration of the S, $\textrm{G}_2$ and M phases takes about 62% of the cycle duration, while the $\textrm{G}_1$ phase takes about 38%^48^.

Considering that the colonies in our dataset are derived from cells coming from 6 different donors, of different age and sex, and that cells were plated at different passages (P0 to P3, see Supplementary Table S1), it is natural to ask how much of the observed variability is linked to these factors. Interestingly, the data show that the degree of heterogeneity within one experiment (same donor, age, sex, passage, plate) is as large as the heterogeneity between different experiments; indeed, it may happen that two cells from the same donor, cultured within the same experiment on the same plate, give rise to two completely different colonies: for example, lineage 20230113_07 has no inactive cells and is perfectly whole, while 20230113_09 from the same experiment is highly broken (see Fig. 2a). In Fig. 4 we report the mean division times $\overline{\tau }$ of all colonies in our dataset grouped by experiments, reporting donor, age, sex and passage. Although we cannot completely exclude some very weak correlation between mean division time, $\overline{\tau }$, and passage (Spearman correlation *ρ =0.30*, with barely significant *P*-value*= 0.046*), no clearcut correlations among the other parameters are observed. We acknowledge, though, that our study involves only 6 donors and therefore a limited demographic diversity, which could not represent the full spectrum of BMSC heterogeneity. Nevertheless, our results suggest that the intrinsic variability within each cell culture experiment largely dominates the overall heterogeneity of the data (Fig. 4).

### Correlation between topology and kinetics

**Fig. 5:**
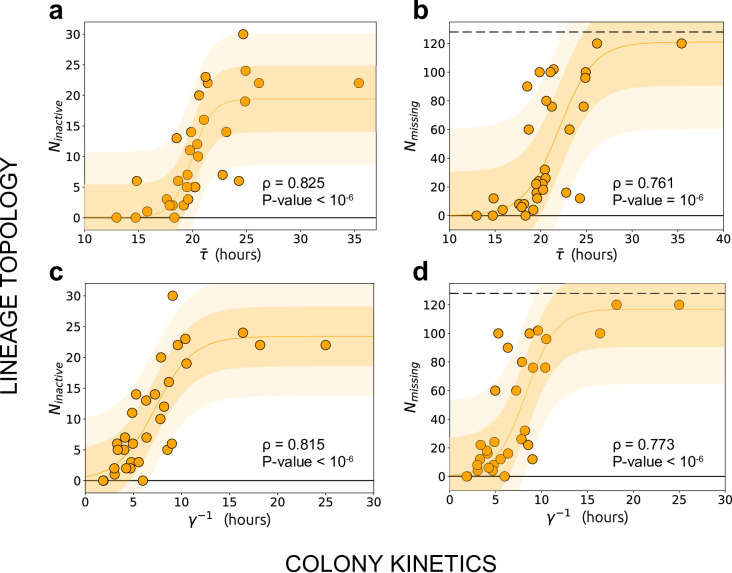
The correlation between topology and kinetics. (**a**) We plot the number of inactive cells of each colony, $N_\textrm{inactive}$ as a function of the mean division time of that colony, $\overline{\tau }$ (orange points). The Spearman correlation between $N_\textrm{inactive}$ and $\bar{\tau }$ is both strong and significant: correlation coefficient *ρ =0.825* and *P*-value $< 10^{-6}$. (**b**) A second characterisation of topology is given by the number of missing cells at generation *k=7*, $N_\textrm{missing}$, which also happens to be strongly correlated to the mean division time, $\overline{\tau }$ (Spearman *ρ =0.761*, P-value $=1.0 \times 10^{-6}$). The dashed line represents the maximum number of missing cells, namely $2^7=128$. (**c**,**d**) The rate *γ* extracted from the fit of the division times histograms is a second characterisation of kinetics. We plot $N_\textrm{inactive}$ and $N_\textrm{missing}$ vs. *1/γ* and find in both cases a strong and significant correlation (*ρ =0.815*, P-value $< 10^{-6}$ and *ρ =0.773*, P-value $< 10^{-6}$, respectively). Shaded areas are just a guide for the eye, corresponding to an amplitude of 1*σ* (darker orange) and 2*σ* (lighter orange) around a sigmoidal fit of the data, where *σ* is the standard deviation of the residuals.

To quantify the previous observation that broken trees correspond to slower colonies, we examine the relation between the total number of inactive cells $N_\textrm{inactive}$ and the mean division time $\overline{\tau }$ (both directly measured from the data) in Fig. 5a: despite the scatter, we find a strong and significant correlation (Spearman: *ρ =0.825*, *P*-value $<10^{-6}$). Such correlation does not depend on the parameters we choose to characterize topology and kinetics: we can use the number of missing cells, $N_\textrm{missing}$, or the inverse proliferation rate *1/γ* (which derives from a fit to equation (1)) and the results do not change: in Fig. 5b, c, and d we show that all these correlations are strong and significant, meaning that the connection between lineage topology and colony kinetics is robust. We emphasize that these two traits are determined by two phenotypically *well separated* sub-populations: topology is uniquely determined by *inactive* cells, while kinetics is exclusively determined by *active* cells. It is therefore surprising that they are in fact strongly correlated phenomena.

In Ref.^22^ it was found that the division time of parents of cells that both divided was shorter than the division time of parents of cells which both stopped dividing, with mixed dividing-not-dividing pairs in between; in Ref^24^ it was reported that cells belonging to slow-dividing progenies had more senescent daughters on average than cells belonging to fast-dividing progenies. Both observations were made at the single-cell level and were based on a binary or ternary classification of the kinetics. Our result shows that the correlation between topology of the inactive cells and kinetics of the active cells remains strong at the level of the entire colony, and that it is robust against unfolding the binary slow-fast classification into the full spectrum of the mean division times.

The strong correlation between the active and inactive phenotypes suggests that the factors regulating the duration of the cell cycle are also involved in the determination of cell cycle exit. This is consistent with the proteomic profiling of BMSC colonies of Refs.^49^. In those studies colonies were classified as fast-growing or slow-growing, depending on the time taken to reach 20 doublings, and it was noted that fast colonies had tripotential differentiation capacity while slow colonies had unipotential differentiation capacity (notice that no lineage was performed in those studies, as tracking individual cells at 20 doublings is simply impossible; the proliferative capacities of the individual clones were approximately assessed in a very crude way according to the time taken to reach confluence in a T75 flask^50^). Proteomic analysis showed that calmodulin (CAM) and tropomyosin (TM) had enhanced expression in *fast* colonies^49^; CAM blocks Fas-mediated apoptosis^51^ and more generally CAM and TM act as modulators during cell proliferation. On the other hand, the over abundance of caldesmon (CAD) and Annexin-I in *slow* colonies was equally relevant, because CAD acts as a regulated break to cytokinesis, while overexpression of Annexin-I reduces cell proliferation^49^. Therefore, proteomic profiling indicates that fast-growing colonies express factors that may reduce the number of cell deaths (although not necessarily of $\textrm{G}_0$cells) and increase proliferation, whereas slow-growing colonies have a larger abundance of proteins potentially depressing proliferation. Our results suggest that some of the factors highlighted in Ref.^49^ may also be involved in triggering the $\textrm{G}_0$ state of BMSCs.

A simple model based on the competition of two mechanisms (one promoting proliferation, with rather large variability in speed, and another, more stable, causing senescence) could explain the observed correlation. A slow but steady buildup of factors precluding proliferation would cause the cell to become inactive unless the proliferation-enhancing mechanism acts first and triggers cell division. If the proliferative mechanism is slow, then there would be a higher chance that the cell becomes inactive. However, the slow-fast classification of Ref.^50^, using 20 doublings, involves a time window *hugely* longer than that of our experiments ($2^{20}>$ one million). While the consistency between our results and those of^50^ suggests that the long-term proliferation potential is already detectable in early generations (*kłe 7*), specific experiments directly comparing the short- and long-term behaviour of individual colonies should be performed to validate such promising connection.

### Synchronization

**Fig. 6:**
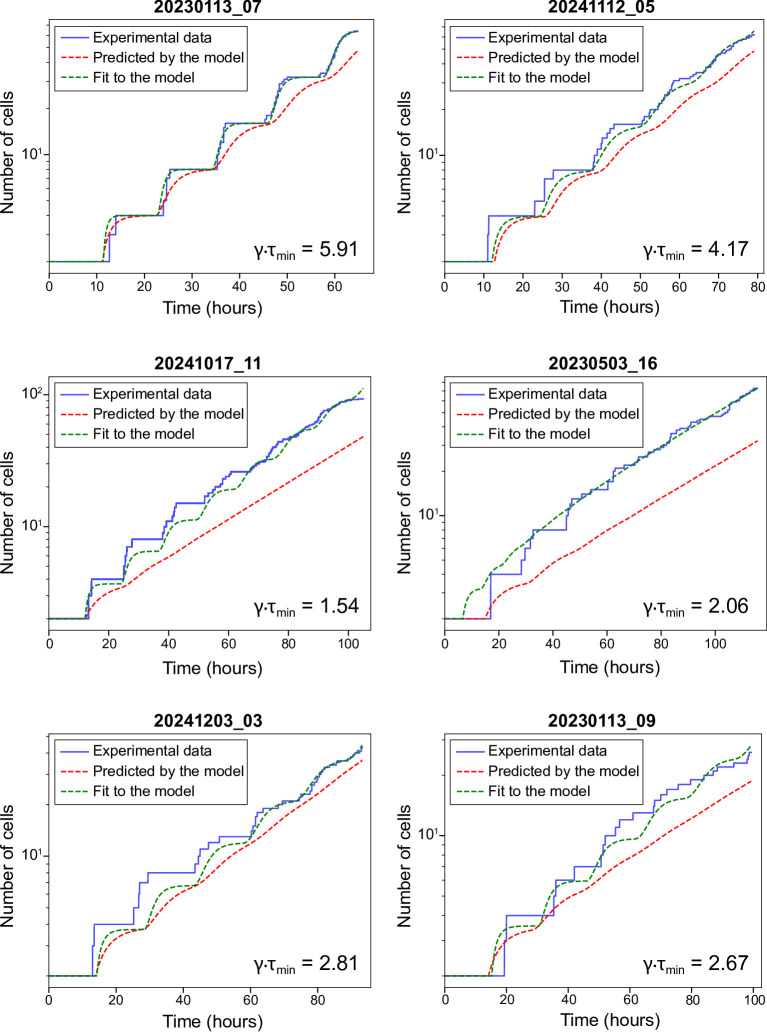
Number of cells vs. absolute time. We show *N*(*t*) vs *t* for the same colonies as in Figs. 2 and 3. A step-like growth of *N*(*t*) is very clear for the colonies with the largest values of $\gamma \tau _\text {min}$ (with *γ* and $\tau _\text {min}$ obtained from the fits to *P(τ )*, see Fig. 3). The steps indicate synchronization of mitosis events; this synchronization is expected even if mitosis are independent events, when the average division time is close to the minimum time, corresponding to $\gamma \tau _\text {min}\gg 1$ (see text). synchronization can be enhanced by the presence of correlations of division times between individuals close in the lineage tree. Blue lines: experimental data; dashed red lines: prediction of the branching process model, with parameters taken from the lineage trees and the fitted *P(τ )*; dashed green lines: direct fit to the branching process form of *N*(*t*).

We now examine the overall growth of the colonies, by measuring the total number of cells at a given time, *N*(*t*) (all growth curves start with *N=2* at *t=0*). This aggregate information is the result of the interplay between kinetics and topology, since how fast the colony grows depends on how fast individual cells proliferate, but also on how many of them remain active. A remarkable feature of *N*(*t*) (Fig. 6) is the presence of steps, a clear hallmark of *synchronization*: when most mitosis occur at approximately the same time, right after the common minimum division time, $\tau _\textrm{min}$, there are “avalanches” of syncronised mitosis (in Supplementary Video S2 we show synchronization of four cousin cells); such synchronization manifests as steps in the growth curve, which mark the transition between different generations, with plateaus at *N= 2, 4, 8, 16, …*. Synchronization of the cells’ mitosis is stronger for earlier generations.

To illustrate this mechanism, we consider the simple case of sister cells. Let us define the *desync*, *Δ t*, as the difference between the times at which the two sister cells divide. If we momentarily assume that there is *no* correlation between the division times of sister cells (we will soon come back to this – wrong – assumption), it is easy to show (see Supplementary Equations) that the mean square desync is given by,

3 $$\begin{aligned} \left\langle (\Delta t)^2\right\rangle = 2 \sigma _{\tau }^2 \ , \end{aligned}$$

where $\sigma ^{2}_{\tau }$ is the variance of the distribution of the division times. The meaning of (3) is quite intuitive: if the division times of the population are all nearly the same, so that their distribution is very narrow (small variance), we indeed expect that all divisions at the same generation occur almost at the same time (small desync); conversely, if the division times are quite different from each other, so that their distribution is broad (large variance), then we do not expect that same-generation divisions are synchronized (large desync). Therefore, in absence of correlation, synchronization depends entirely on how narrow is the distribution of the division times. But in order to observe sharp steps in *N*(*t*) it is not enough that the ramp between two plateaus is short: it must be much shorter than the length of the plateaus themselves, namely the desync must be much smaller than the average cycle length, $\sqrt{\left\langle (\Delta t)^2\right\rangle } \ll \langle \tau \rangle$. From equation (3), we conclude that steps will be sharper when $\sigma _{\tau } \ll \langle \tau \rangle$. Synchronization will be slowly lost as generations progress, due to random fluctuations in division times, and steps will die out.

For the gap-exponential distribution that we have used to fit our data, Eq. (1), the variance is connected to the proliferation rate through the relation, $\sigma _{\tau } = 1/\gamma$, while the mean is given by, $\langle \tau \rangle = \tau _\textrm{min}+\gamma ^{-1}$, where we recall that $\tau _\textrm{min}$ is the minimum division time. We conclude that (under the assumption of uncorrelated division times) the steps of *N*(*t*) will be sharp when $\gamma ^{-1} \ll \tau _\textrm{min}+ \gamma ^{-1}$, that is (multiplying by *γ* left and right) $1 \ll \gamma \tau _\textrm{min}+1$, which can only be verified if,

4 $$\begin{aligned} \gamma \tau _\textrm{min}\gg 1 \ . \end{aligned}$$

This prediction is qualitatively consistent with the experimental growth curves *N*(*t*) and with the parameters of *P(τ )* from the gap-exponential fit: the largest values of $\gamma \tau _\text {min}$ are found for colonies 20230113_07 and 20241112_05, which have the sharpest steps.

### Branching process models

Qualitatively explaining the mechanism of sharp vs smooth steps in the growth curves may seem a validation of the assumption of uncorrelated division times; however, *N*(*t*) must be *quantitatively* accounted for by a satisfactory model. We now ask whether an uncorrelated model, in which cells divide independently from each other, is actually up to this task. Models of this kind are called *branching processes*^37^ and their input is the probability distribution of the division times, *P(τ )*; once this is specified, one can derive the growth curve, *N*(*t*).

If one employs as an input an exponential distribution *P(τ )* with no gap, one gets an exponential growth of *N*(*t*), with no steps. But the actual distribution of the division times *does* have a gap, $\tau _\textrm{min}$. Using the gap-exponential *P(τ )* as an input of the branching process one obtains that *N*(*t*) grows in (rounded) steps^39,42^, not unlike those we observe here. However, in the model studied in Ref.^42^ all cells were active, while inactivity is a crucial ingredient of BMSC phenomenology. Therefore, we have generalised the model to include inactive cells: death is modeled through a probability that at the end of its life the cell is removed, while $\textrm{G}_0$ cells are modeled through a probability of having an infinite lifetime (see the Supplementary Equations for details). This new model has four parameters, which can be estimated from the experiments: *γ* and $\tau _\text {min}$ from the fit to *P(τ )* (Fig. 3a), plus the death and $\textrm{G}_0$ probabilities, which can be extracted from the death/$\textrm{G}_0$ fractions in each tree. This model’s predictions for *N*(*t*) are shown as dashed red lines in Fig. 6: the agreement is poor; in particular, the synchronization steps are much blunter in the model than in the experiments. If, instead, we perform a least squares fit of the theoretical model to the experimental *N*(*t*), we obtain the dashed green curves in Fig. 6. In this case the agreement is better; however, even though the fit yields a value of $\tau _\text {min}$ rather close to the experimental one, the fitted value of the proliferation rate *γ* is systematically larger than its experimental counterpart; hence, the better agreement with this second procedure is only apparent, because it implies an incorrect *P(τ )*.

We conclude that the proliferation of BMSC colonies is much more synchronised than it would be expected from an uncorrelated branching process characterized by a distribution of division times equal to the experimental one; conversely, if we insist on quantitatively accounting for the observed synchronization with an uncorrelated branching process, we end up with a distribution of the division times that is much narrower than the experimental one. What is the origin of this discrepancy?

### Correlations and the failure of branching process models

The branching process model makes a key assumption, which is also at the basis of equation (3), namely that the division times of different cells are uncorrelated. However, positive correlations between the division times of sister cells are known to exist and have been reported since at least the 1950s^27^ in several kinds of cells^41,52–54^. Correlations can enhance synchronization, therefore giving more pronounced steps in the growth curve for a given division time distribution.

Let us go back to the square desync between sister cells, but this time let us *not* assume that their division times are uncorrelated. Some simple algebra (see Supplementary Equations) shows that in this case the square desync is given by,

5 $$\begin{aligned} \left\langle (\Delta t)^2\right\rangle =2 \sigma _\tau ^2 \left( 1 - \rho _\textrm{P}\right) \ , \end{aligned}$$

where $\rho _\textrm{P}$ is the Pearson correlation coefficient between the sisters’ division times. If $\rho _\textrm{P}=0$, we go back to Eq. (3); but we see that the existence of positive correlation, $\rho _\textrm{P}>0$, has the effect of decreasing the desync, hence *increasing* synchronization, even at fixed $\sigma _\tau$. It is important to appreciate these two different sources of synchronization, namely the low overall variance of the division times and the large correlation of the sisters’ division times: even when the variance is large, a strong correlation, $\rho _\textrm{P} \sim 1$, would give near-perfect synchronization, as cells’ division times would all strongly fluctuate, but they would do that *together*. If cells are not sisters other terms enter into play in the equation, leading to gradual desynchronization as generations go by, but the effect of correlations between the division times of cells belonging to the same generation is still that of increasing synchronization. Therefore, positive correlation of same-generation division times – which the branching process model neglects – enhances synchronization. The discrepancy between experiments and simple branching process theory therefore indicates that equal-generation division times in BMSCs are positively correlated. A further support of this interpretation is provided by the relatively poor job done by the model fitted on the first 7 generations, to predict the behaviour of *N*(*t*) up to generation 8 (see Supplementary Fig. S4 for this prediction in the three fastest colonies).

There is a second tacit assumption of the branching process, namely that the distribution of division times, *P(τ )*, is the same for all generations; this hypothesis may also be inadequate. We have computed the average division time at each generation, $\overline{\tau (k)}$ (see Supplementary Fig.S5); in line with previous findings^22^, $\overline{\tau (k)}$ shows a weak increasing trend, pointing to a generation-dependent $P_k(\tau )$. The increase of the average means that our *P(τ )*, which is consolidated over all generations, is broader than the distributions at fixed *k*, which may be a further reason why the branching process fails quantitatively.

To summarize, we have shown that simple models of cell proliferation, in which cells divide independently of one another, cannot simultaneously reproduce the statistics of division times and the level of synchronicity displayed by the growth curves. This finding points to the presence of positive correlations between division times within the same generation. Further analysis should quantitatively and systematically measure these correlations, incorporate them into a modeling framework, and possibly elucidate their origin. In this respect, we note that it is unlikely that cell-cell contact and density play a role. Indeed, we measured the median distance between sister cells at the end of their life cycle and we found *d=0.43*mm (i.e. of the order of 10 body-lengths). This means that sister cells are not in physical contact or proximal to one another when they divide. A more sophisticated mechanism should be at play. Work in this direction is underway.

## Conclusions

By conducting time-lapse microscopy on a set of 32 single-cell-derived colonies of human BMSCs, we discovered a sharp correlation between lineage topology (which is determined by the sub-population of inactive cells), and colony kinetics (which depends entirely on the division times of the sub-population of active cells): lineages that are broken by the presence of many inactive cells (typically $\textrm{G}_0$) are also characterized by an active sub-population with a slower proliferation rate and *vice-versa*. This correlation connects a binary state (active-inactive) to a continuous variable (division time) that depends on the molecular expression. Our study also reveals the presence of kin correlations between the division times of same-generation cells, which manifest in the degree of synchronization of population growth.

As it happens with many other traits of BMSC colonies, both the number of inactive cells and the mean division time of active cells —as well as other quantities characterizing topology and kinetics— are strongly heterogeneous. The interesting consequence of having found a sharp correlation between topology and kinetics, is that it reduces the number of potential *independent* factors at the origin of the heterogeneity of both traits, hence partly reducing the complexity of the problem. By establishing a mutual dependence of the fluctuations of the number of $\textrm{G}_0$ cells and the fluctuations of the division times, we effectively turn what seemed independent random variations, into a phenomenon with a single underlying causal factor. This factor needs to explain why, within the same clonal cell population, a longer cell cycle is associated to an increase of the probability to enter the $\textrm{G}_0$ state.

Our findings provide fundamental biological insights about human BMSCs: we demonstrate that the propensity of individual BMSC colonies to produce senescent or apoptotic cells cannot be deduced from the characteristic of the donor, as the mean division time of the colonies is not correlated with donor sex and age and weakly correlated to the number of passages in culture. Additional experiments with a larger number of donors are required to precisely assess the impact of age and sex on the BMSC growth kinetics. Rather, our findings show that it is the number of inactive cells in individual BMSC colonies that strongly correlates with the proliferation properties of the dividing cell fraction, pointing at the kinetics of growth as a potential selection criteria for *in vivo* application.

The heterogeneity seen in BMSC colonies may reflect the presence of cells with different commitments, from very undifferentiated SSCs, to committed progenitors with osteogenic or adipogenic capacity, to cells with limited differentiation capacity like fibroblasts. Further studies are needed to understand if different growth patterns reflect different differentiation capacity of BMSC clonal populations. In Ref.^9^, colonies of BMSCs were transplanted into immunocompromised mice to determine the bone-forming capacity of these colonies and assess the correlation between this capacity and their proliferation rate. Although kinetics was not found to be strictly predictive of osteogenesis, weak correlation between proliferation rate and bone formation *in vivo* was anyway found. A subsequent study^10^ distinguished between colonies that upon *in vivo* transplantation formed fibrous tissue, those that formed bone, and those that formed bone/marrow organ, where only this third class was deemed multipotent. However, the proliferation rate and its possible correlation with multipotency were not assessed *in vivo*. Therefore, although it seems that high proliferation rate and hence a faster overall kinetics —which in turn implies a smaller number of inactive cells— may be positively correlated to the multipotency of BMSCs (likely enriched in SSCs), new *in vivo* studies are needed to correlate kinetics to *bona fide* multipotency.

Finally, the strong heterogeneity observed among BMSC colonies suggests that clonal expansion could represent a useful experimental strategy to identify subpopulations with distinct growth pattern and proliferative capacities. Although we do not assess long-term proliferation capacity in our study, previous reports have shown that only a small fraction of single-cell–derived BMSC colonies can be expanded long-term^55^, indicating that high proliferative capacity is not a universal feature of BMSCs. While the selection of highly proliferative clones may therefore be promising for regenerative applications, more specific kinetics and growth patterns of these clones must be defined, since it is well established that prolonged *in vitro* expansion can compromise multipotency and *in vivo* bone-forming capacity^56^. Consequently, future studies will be required to directly assess which clones retain robust osteogenic potential *in vivo*, and to determine the extent to which clonal selection strategies can be translated into therapeutic bone regeneration.

## Supplementary Information

Supplementary Information 1.

Supplementary Information 2.

Supplementary Information 3.

## Data Availability

The datasets generated in the current study are available from the corresponding author on reasonable request.
