# Supplementary material for: Lineage topology, replication kinetics and cell cycle synchronization reveal regulated growth dynamics in human bone marrow stromal cell colonies
Source: Sci Rep. 2026 Jun 4;16:25408. doi: 10.1038/s41598-026-51809-z (PMC13473582; doi:10.1038/s41598-026-51809-z)
Supplement: Supplementary file 1 [file 41598_2026_51809_MOESM1_ESM.pdf]

Lineage topology, replication kinetics and cell cycle synchronization reveal regulated growth dynamics in human bone marrow stromal cell colonies

Supplementary Methods

Cell source

Human bone marrow stromal cells (BMSCs) were isolated from bone samples harvested from healthy subjects undergoing elbow orthopaedic surgery through procedures approved by the local ethical board (Rif. 5313 Prot. 387/19) and upon informed consent of the patients. Bone fragments were collected in Minimum Essential Medium with α modification (α-MEM, Merck, Saint Louis, USA) supplemented with 1% Penicillin/Streptomycin (P/S, Merck) by the surgeon and transferred to the laboratory where the isolation of the cells was performed. Bone pieces were minced and washed with phosphate-buffered saline (PBS) in order to allow the release of bone marrow cells. Cell suspensions were collected in culture medium consisting of α-MEM supplemented with 20% Fetal Bovine Serum (FBS, Thermo Fisher Scientific, Waltham, USA), 1% P/S and 1% L-glutamine (L-gln, Merck) and counted on a hemocytometer. Cells were cultured at a density of $1\text{–}20 \cdot 10^3$ nucleated cells/cm^2 at 37°C, in a 5% CO_2 atmosphere. The remaining cells were cryopreserved at the cell density of $1\text{–}3 \cdot 10^7$ total cells (including red blood cells) per mL of Bambanker Serum-free cell freezing medium (Nippon genetics Europe) for subsequent uses.

Cell planting and seeding density

After 24 hours of culture, non-adherent bone marrow cells were removed and adherent BMSCs were grown as P0 until confluency of the colonies (10-14 days). Confluent BMSC colonies were detached using 0.05% trypsin-EDTA (Merck), counted and plated as P1. The same procedure was applied to establish P2 cultures. Time lapse experiments were performed with either P0 to P3 cultures (see Table S1). P0 experiments were performed with freshly isolated BMSCs. P1 experiments could be established either starting from freshly isolated cells, or from cryopreserved bone marrow cell suspensions. P2 cultures were established starting from cryopreserved bone marrow cell suspensions. To prepare the cultures for time lapse experiments, cells were plated at a density of 50 cells/dish in 35 mm μ-Dish Petri, model 81156 (Ibidi, Gräfelfing, Germany). The culture medium was filtered through a 0.22 μm syringe filter to prevent the presence of serum residues in the culture. Two identical dishes were generated, one starting the time-lapse experiments in the microscope stage top chamber incubator, and the other incubated in the standard incubator (Heracell, Heraeus) as positive control of cell growth. After 6 hours, cells in the two dishes were checked to verify their attachment, and one of the two dishes was inserted in the microscope to start the time-lapse imaging, while the second dish becomes the control (see below). At this stage, the actual number of cells found in the observational area of the μ-Dish ranged between 10 to 20.

Time-lapse equipment

The microscope is a phase contrast Nikon Inverted Eclipse Ti2-U, controlled by Nikon NIS-Elements Advanced Research software version 5.30. The microscope is equipped with a Nikon 4X objective MRH20045, a Nikon 10X objective MRH20105, and a Nikon 20X objective MRH48250. The images we use to perform the data analysis are taken with the 20X objective, but we also use the 4X objective for some auxiliary tasks. The camera is a Nikon DS-Qi2 monochrome 14 bit with a resolution of 16.25 megapixels. Within the microscope there is an Okolab (Pozzuoli, Italy) stagetop chamber incubator, H301-NIKON-TI-S-ER, equipped with a two-well base H101-2 X 35-M, designed to host two dishes. The chamber incubator is mounted on the motorized translational stage Nikon Ti2-S-SS-E, thanks to which we could monitor several colonies in parallel within the same dish. The gas mixture is controlled by an Okolab Bold-Line module H301-T-UNIT-BL-PLUS (Okolab-BL). Redundant measurements of temperature and CO_2 are performed with an Okolab-LEO module.

The time lapse sampling period of experiments `20230113` and `20230503` was 20 min, while the sampling period of experiments `20240627`, `20240924`, `20241017`, `20241112`, `20241203`, `20250313`, `20250328`, `20250612` and `20250626` was 15 mins. This change in the sampling period was due to the need to simplify and therefore speed up the tracking procedure.

Culture control during time-lapse

The conditions of the cell culture during time lapse were standard, namely humidity 90%, CO_2 5%, temperature 37°C. *Gas control:* The gas mixture of air, water and CO_2 was produced and monitored by the Okolab-BL module; water to provide humidity was filtered and autoclaved. To have an independent control of the CO_2 concentration within the incubator, we

measure and store it every 5 minutes through the Okolab-LEO module: gas is pumped out of the incubator into the LEO for 3 minutes, so that an independent reliable determination of the CO_2 concentration can be made. In the months before the experiment started, we calibrated this double-monitoring system in such a way to have consistent readings within the same incubator. *Temperature control:* The incubator hosts two dishes. The first dish is the one that we use for the time-lapse, seeded with BMSCs, in which there are no temperature probes, in order not to perturb or contaminate the cells; the second dish, at 6 cm from the cell-seeded dish, is used exclusively for temperature control (the Temperature-Control Petri, T-CP). In the T-CP there is an amount of sterilized SIGMA water identical to the amount of medium in the cell-seeded dish (2 ml). Within the T-CP there are two temperature probes; the first one is used by the Okolab-BL module to fix the temperature at 37 degrees through a digital feedback; the second probe is connected to the Okolab-LEO module that independently records the temperature every 5 min. In the months before the experiment started, we calibrated this double-monitoring system in the following way: we located the T-probe connected to the Okolab-BL module within the T-CP and the T-probe connected to the LEO within the dish that would contain the cells in a real experiment, and checked that the two readings were consistent; during this testing period the discrepancy between the two temperature probes was always lower than 0.2°C.

Selection of the originating cells

Six hours after the primary dish is seeded with cells, the dish is inserted into the microscope incubator. The first thing we need to do is to identify a couple of cells in the dish in order to tune the focus of the 4X objective; this procedure (which needs to be performed in live imaging) never takes more than 10 minutes, and often significantly less. When the focus is fixed, we scan the entire dish; the scan is performed by taking $8 \times 8 = 64$ photos covering the entire dish. The image resulting from this scan is then used to select the originating cells to monitor during the experiment (see below). It is very important that this selection phase (which may be long, typically up to 30 minutes) is not performed in live imaging with the microscope lamp on, because that would dramatically increase the danger of phototoxicity (see below). By working on the scan photograph, we can leave the cell-seeded dish dark. Nevertheless, it is advisable to keep the selection procedure as quick as possible to avoid that potentially isolated cells candidates start dividing before the time-lapse is initiated.

Within this study we only consider BMSC colonies that are derived from a single cell (single-cell-derived colonies in the classification of [1]). Very few studies have demonstrated unequivocally that the analyzed colonies were derived from single cells: micro-wells are checked typically for the presence of a single colony days after plating, but this protocol does not verify that these isolated colonies each started from a single cell, and a colony that originated from two cells in close proximity has high likelihood of exhibiting heterogeneity. Here, we cut to zero this risk by only selecting at the beginning of the experiment single cells that are completely isolated, so that we are sure of the true clonal nature of each one of our colonies. Moreover, we only monitor colonies that are not infiltrated by other colonies during the time-lapse.

To meet these two requirements we seed the Petri dish at very low densities (see above), in order to have a higher probability to find isolated originating cells. The fact that the initial cell must be isolated has a twofold importance: first, by selecting an isolated cell we give to the colony generated by that cell the necessary space to proliferate without interfering with other colonies during the time duration of the experiment; hence, here by 'isolated' we mean that the distance from another originating cell must be of the same order as (or larger than) the average size of the colonies at generation $k = 7$. Secondly, by selecting only isolated cells, we are sure to avoid cases in which we have two (or more) cells close to each other at the beginning of the experiment, because in that case we cannot be sure whether the nearby cells are sisters or not, which is essential to define single-cell-derived colonies. Given the very low seeding density, the size of our dish, and the size of the colonies at $k = 7$, the isolation criterion severely limits the number of colonies we manage to successfully select and follow during each experiments; this is the chief reason why our experimental campaign lasted for so long. We never selected more than 20 initiator cells, only a handful of which generate colonies that are finally included in the study (see below).

Once the cells have been selected from the scan image, we switch to the 20X objective and fine-tune the focus for each one of the selected cells, in order to optimise the sharpness of the images; moreover, in this phase we tune the position of each selected originating cell in order for it to be precisely at the centre of the field of view. This procedure is quick (max 10 min) and needs to be done in live imaging, with the lamp on. Finally, after the focus of the 20X objective is set, we start the time lapse.

Monitoring the experiment

The time-lapse experiment is monitored every 6 hours for its whole duration, according to the following check-list: (i) the temperatures measured by the two probes within the T-CP must be consistent with each other and both equal to 37 ± 0.5°C; (ii) the graph in the past 6 hours of the temperatures measured by the two probes within the T-CP must show no anomaly; (iii) the CO_2 measured by the BL module at the gas source and by the LEO module within the incubator must be consistent with each other and equal to $5 \pm 0.2\%$; (iv) the graph in the past 6 hours of the CO_2 measured by the same two modules must show no anomaly; (v) all photos must be sharp and all cells must be well in focus; (vi) colonies are checked for the need to expand the field of view (see below); (vii) colonies are checked for infiltration (see below); (viii) a rough count of all cells within each colony is performed. Every 24 hours a scan of the entire dish at 4X is performed, to have a global view of the

development of all colonies in the dish. Since colonies' kinetics strongly fluctuate, the total experiment duration is variable: single experiments lasted between 10 to 14 days. The time at which a colony reaches the seventh generation can be as short as 5 days for fast colonies but significantly larger for slower colonies.

Field of view expansion

The field of view (FOV) around each colony is composed by $n \times n$ photographs at 20X, acquired by translating the chamber incubator with the motorized stage and stiched together by the proprietary Nikon software NIS-Elements. At the beginning of the time lapse, when there is only the originating cell, we set $n = 3$. As the colonies grow it becomes necessary to expand the FOV, hence $n = 3, 5, 7, \ldots$. We always use an odd value of n so that the FOVs before and after the expansion have the same center; this simplifies subsequent image analysis. Our current version of the software does not allow to selectively expand the FOV of only certain colonies, therefore a FOV expansion from n to $n+2$ implies a significant increase of the total number of photographs taken at each iteration. This introudces some constraints related to phototoxicity that we discuss below.

Criteria to stop monitoring colonies

During the experiment we can stop monitoring a colony for one of the following reasons: (i) Despite the isolation criterion, a colony may come in contact with another colony during the experiment (infiltration); in that case we stop monitoring and we discard it for data analysis. The infiltration criterion is the following: colonies A and B are mutually infiltrated if the distance between the two closest cells of A and B is equal to or smaller than the mean inter-cell distance within A and B. (ii) A selected originating cell may either die or detach from the dish before starting mitosis; in this case we stop monitoring it. (iii) When a selected originating cell does not make the first division within 4 days, we stop monitoring it. (iv) If one or more cells belonging to a colony drop out of the FOV before we manage to expand it, we stop monitoring that colony. As a general rule, we keep monitoring all colonies that do not incur in the criteria above in order to avoid any selection bias. In some cases, however, it becomes necessary to forcibly reduce the number of monitored colonies. This is because as the colonies grow we need to expand the FOV, but as we shall see later on, the total number of photographs for each loop in the time-lapse is limited to 750 to avoid phototoxicity effects. Hence, when FOV expansion is absolutely urgent but would exceed the total of 750 photographs and no colonies match the discard criteria above, we drop one colony randomly. Finally, of all colonies monitored during the experiment, we only include in our analysis those that produce *at least* two cells at generation $k = 7$, lest the seventh generation criterion we have adopted to characterize the whole study becomes void.

We notice that by adopting the isolation criterion we are not biasing towards a particular distance, but we are simply imposing a minimal distance between originating cells, in such a way that all analyzed colonies grow in the same standard conditions of isolation. It is possible that colonies growing not in isolation, but rather in full merging with other colonies nearby could behave differently; but including these colonies in the dataset would introduce an unacceptable degree of arbitrariness, because while there is just one way to define a single-cell-derived colony, there are endless ways in which different colonies can grow and proliferate in contact with each other, so that one would be adding heterogeneity in the data set, which is merely derived from a poor experimental setup.

Medium replacement

Medium is changed every 3–4 days (typically every Tuesday and Friday, with the experiment starting on Friday), for the whole duration of the experiment. Whenever the medium is changed in the cell-seeded dish, the sterile water is also changed in the T-CP, so to have always the same amount of fluid in the two dishes, which is essential to have a reliable temperature control.

Illumination parameters and phototoxicity

To obtain brilliant and sharp images (which immensely simplifies the chain of analysis downstream) one would ideally illuminate the cells as much as possible during imaging. However, this is not advisable due to the grave danger of phototoxicity. In order to select the illumination parameters (light intensity, filters, exposure time) it is therefore essential to keep into account the potential phototoxic effects of a prolonged exposure of cells to light.

Phototoxicity is a very dangerous effect, which acts in two ways: directly, by interfering negatively with the various cellular processes, among other things depressing proliferation and increasing the apoptosis rate [2–7]; and indirectly, by altering the properties of the culture medium [8]. Phototoxicity can severely bias the data and must therefore be kept strictly under control. The issue of phototoxicity is very complex: experimental tests are few and conducted on rather diverse cell types, by observing different cellular traits; hence it is difficult to establish a consensus on the danger threshold of light level from the data in the literature.

The chief parameter to assess the impact of phototoxicity is *fluence*, defined as the energy per unit area hitting the culture surface, measured in Joule per square centimetre (J/cm^2). In BMSCs, the reported critical value of fluence above which clear effects on cellular processes are observed ranges from $21\,J/cm^2$ [2] to $12\,J/cm^2$ [5], but the danger threshold has been estimated as low as $4\,J/cm^2$ in ASC cells [6]. Regarding medium degradation, the most recent experiments indicate a toxicity threshold of

21 J/cm^2 [8]. From these results and given the lack of information about fluence and illumination parameters in most, if not all, time-lapse studies of BMSCs, we chose to stay well below the lowest of the above-mentioned bounds, namely 4 J/cm^2. To do that, we proceed as follows.

In all our experiments the light intensity of the LED lamp of the microscope (Cree, Inc. XM-L2 series, `XLMBWT-00-0000-0000T60E2`) is reduced by 5 stops (i.e. a factor $2^5 = 32$) by means of a neutral filter. By using a power meter (Thorlabs PM200, sensor Thorlabs S170C), we measured the power of the filtered light; even though the power does not depend on the objective, each objective requires a different phase filter to work, and the intensity of the light received by the sample *does* depend on the phase filter. Hence, all power measurements must be performed with the appropriate phase filter. To compute the power, the meter needs as an input the wavelength of the LED light. Our LED's spectrum has two primary peaks (at 440 nm and 540 nm); to be conservative in our estimates of the fluence we input the power meter with the wavelength of the most energetic peak (440 nm). Power is the energy per unit time (milliwatt, mW), hence by dividing the measured power by the area of the disc of light produced by the lamp (0.64 cm^2) we obtain the power per unit area that the sample receives, called *irradiance* (notice that for this to be true the light disc area must be smaller than the sensor area, which is the case in our setup; otherwise the power must be divided by the sensor area). Irradiance is measured in mW/cm^2. Once the irradiance is determined, the fluence —total energy per unit area— is obtained by multiplying the irradiance by the exposure time. The exposure time depends on the specific phase of the experiments; those below are the values of the illumination parameters and relative irradiance and fluence during each one of the phases when the microscope lamp is on: (i) *Initial cell search* — Once the cell-seeded dish is inserted into the microscope incubator we first need to identify a couple of sample cells, in order to tune the focus on them. In this phase, the light intensity is set at 40%, the 5-stop filter is on, and we use a 4X objective, giving an irradiance of 0.0086 mW/cm^2; the camera sensor gain is set at 1.0X. The search lasts a maximum of 10 minutes (often significantly less), giving a fluence smaller than 0.006 J/cm^2. This operation is done once in the experiment. (ii) *Scan of the Petri dish* — We scan the cell-seeded dish at the beginning of the experiment in order to identify candidate cells; moreover, we scan every 24 hours the dish to have a global view of the experiment's evolution. During this task, the light intensity is set at 4%, the 5-stop filter is on, and we use a 4X objective, giving an irradiance of 0.70 μW/cm^2; the camera sensor gain is set at 1.0X. The scan is performed by taking $8 \times 8 = 64$ photos, and the exposure time of each photo is 2 s, giving a fluence of 9.0×10^{-5} J/cm^2. This operation is done every 24 hours. (iii) *Focus fine-tuning* — Once the originating cells are selected we fine-tune the focus for each cell. In this phase the light intensity is set at 75%, the 5-stop filter is on, and we use a 20X objective, giving an irradiance of 1.0 mW/cm^2. The procedure lasts a maximum of 10 minutes, giving a fluence smaller than 0.6 J/cm^2; the camera sensor gain is set at 4.1X. This operation is done once in the experiment. (iv) *Time lapse* — During the time-lapse the light intensity is set at 75%, the 5-stop filter is on, and we use a 20X objective, giving an irradiance of 1.0 mW/cm^2. The exposure time of each photo is 80 ms, corresponding therefore to a fluence-per-photo of 8×10^{-5} J/cm^2; the camera sensor gain is set at 4.1X. At each iteration the system takes $n \times n$ photos around each colony, where $n = 3, 5, 7, 9, \ldots$. If we are following C colonies, the total number of photos at each iteration is n^2C; we always keep this total number below 750 (in the most flourishing experiments, we may have 6 colonies, each one covered by a 11×11 photo tiling, giving a total of 726 photos at each iteration). In this way we have a maximum fluence of $8 \times 10^{-5} \times 750 = 0.06$ J/cm^2 for each iteration. The whole loop repeats after 15 minutes. We therefore have a time-lapse fluence that is 66.6 times lower than the most conservative bound found in the literature (0.06 J/cm^2 vs. 4.0 J/cm^2). Hence, we are confident that our observations of BMSCs are not affected by phototoxicity.

During preparation, and for the entire duration of the time-lapse, all lights in the lab are off, including all instruments lights and displays; windows and doors are shielded.

Control dish

When the cell-seeded dish for time-lapse microscopy is prepared, a second dish with identical procedure and seeding is also prepared and kept in a different (large) incubator (Heracell, Heraeus) as a control; this is the Control Dish (CD). The CD is not exposed to any light (besides the room and hood lights during medium changes), nor perturbation, and it undergoes medium replacements at identical intrevals as the time-lapse dish. At the end of the experiment we perform a comparative analysis of the dish used for the time-lapse vs. the CD, including a 4X scan of both. In this way we can be certain that there are no statistically significant differences between the two dishes, and therefore conclude that the culture conditions were identical, so that the time lapse procedure has not interfered with the proliferation of the cells. This check is of great importance, especially in connection with the risk of phototoxicity: since this risk is negligible for the CD (which is kept in the dark), comparable proliferation rate and vitality of the two dishes is a further test that no phototoxic effects are in action. All experiments used in this study passed this test.

Cell tracking method

To perform the tracking, a C/C++ program was developed, based on the OpenCV computer vision library [9]. Each of the 32 colonies was semi-manually tracked, requiring an average of 13 to 15 hours of work per colony. The tracking program has a

graphical user interface consisting of two windows: the first displays the acquired images and the second shows the lineage tree that is progressively formed during the tracking process. Since all colonies are single-cell derived, the first step of the procedure is to tag the originating cell in the first frame of the acquisition by mouse-clicking on it; in that way the starting root link of the colony's lineage tree is generated. The originating cell is special in the lineage, because we have no information about the mitosis generating it, so no division time is associated to the originating cell. The next step is to connect the generating cell to its two daughter cells: the originating cell is selected in the lineage tree, which associates a label to that cell; the operator then scrolls through the sequence of images following the cell until reaching the images showing the initiation of the mitosis.

It is important to realize that the tracking procedure is not only very time-consuming, but it also becomes very hard to perform beyond generations 7-8, no matter how much time is employed. The reason is that in some cases cells form clusters, in which the proximity is such that it becomes hard to identify and follow the cells' identities (see Fig.S1). When a cell i enters such cluster, hence temporarily losing its identity, the operator has to suspend the assignment of the identity, since *all* cells in the cluster are potential candidates for the future of i. Using an iterative process, the operator follows *all* cells in the cluster forward in time and then back to the past, maintaining cell identities through the ambiguities using morphological features and dynamic predictions. This process is extremely time-consuming, though, so this is one of the most severe bottlenecks of tracking. Moreover, no matter how much time one invests, beyond generation 7-8, the identity ambiguities become so frequent (due to the large cellular density), that the right identity cannot be sorted out in 100% of the cases. These are the reasons why in the current study we stop tracking at generation seven.

Identifying the mitosis

The precise determination of the division times is crucial for the analyses conducted in this study, hence it is essential to use a well-defined marker of mitosis to uniquely identify a precise moment during the mitosis phase of the cell cycle. Mitosis consists of several phases of different durations; the most distinctive and shortest is the mitotic cell rounding [10, 11], during which the cell assumes an almost spherical shape, appearing as a bright, circular object in the image (see Fig. 1). This trait is easily recognisable and was chosen as marker of mitosis. The same criterion has been adopted in other studies [1]. Clicking on the image corresponding to the mitotic rounding sets the stopping time of the mother cell and the starting time of the two daughter cells; these time-stamps are used to determine the division time τ. At the frame corresponding to the mitotic rounding the two daughter cells are still not distinguishable, hence the operator scrolls on through the sequence of images until the nuclei of the two daughters are clearly separated. At that point the operator clicks on the two daughters, thus assigning a new label to each of them; these labels are connected to the mother's label by the corresponding mitosis. The process is repeated recursively for all cells, until the seventh generation is reached. Cells of the seventh generation are no longer followed, so they only have the starting time, hence we do not have the division times of the cells of generation $k = 7$.

One may ask why we use the cell rounding as marker of mitosis, instead of simply using the same frame as we use to label to two daughters. The answer is that deciding what is the first image at which the two daughters' nuclei are clearly separated from each other has a large degree of arbitrariness and is operator-dependent; therefore, it is not advisable to use this frame as a marker of mitosis (for safety, we anyway checked that these two times are perfectly correlated to each other, see Fig.S6). In contrast, the arbitrariness in the selection of the first image in which the two daughters are well-separated has no consequences on any observable, as it is only used to assign a label to the two daughters.

Determination of the P-value

To assess the significance of the correlation between two sets of M variables, $\{x_i\}$ and $\{y_i\}$, with $i = 1, \dots, M$, we proceed as follows. First, we calculate the Spearman correlation coefficient ρ on the actual experimental variables. Then we randomly scramble the positions of the values in the first set, $\{x_i\}$, hence destroying correlations between the two variables, and we recalculate ρ. We repeat the random scrambling 10^6 times and calculate the fraction of cases that give a correlation coefficient ρ larger than or equal to the experimental one; this fraction is the P-value of Spearman correlation. Notice that with this procedure the most significant correlation that we can get has P-value less than 10^{-6}. The P-value defined in this way is therefore equal to the probability that the correlation found in the data is accidentally present also in an uncorrelated random dataset of the same size and of the same nature as the experimental one.

Supplementary Notes

Robustness of the G_0 criterion

During tracking, two special cases can be met: (1) A cell commits apoptosis and dies; this process is very clear in the imaging, hence there is no ambiguity in its identification. The cell is tagged as 'dead' in the lineage tree and graphically indicated with a red dot. (2) A cell stops dividing, going into the G_0 phase. We have defined a G_0 cell as a cell that has not divided for a period of 84 hours since its birth; when this condition is met, we stop tracking that cell and we tag it as G_0 in the lineage tree. In only three cases we were not able for technical reasons to follow a G_0 cell up to the full 84 h criterion: in colony `20241112_02`

one cell was tagged as G_0 after 68 h; in colony `20250313_14` two cells were tagged as G_0 after 57 h. The morphology of these cells, though, was very typical of the G_0 state. Moreover, all three cells belonged to the last full generation, $k = 6$, hence even in the unlikely case that their G_0 status changed, the topology of their trees would not be significantly altered.

The topology of a given tree entirely depends on the number and distribution of inactive cells; but while dead cells are unambiguously identified, G_0 cells (which are 95% of inactive cells) are less easily so. In our study we have employed a reasonable but still arbitrary operational definition of what a G_0 cell is. We must be sure that by changing the threshold t_c we do not produce trees with significantly different topologies. This objection is not pedantic: if the threshold time is chosen too close to the bulk of the division times of the population, we would mistakenly classify as G_0 cells that are not, and consequently we would produce lineage topologies heavily dependent on the arbitrary value t_c. In order to know that we are not running this risk, we cannot limit ourselves to noticing that 84 hours is much larger than the mean division time of 20 hours; precisely because of the great heterogeneity of BMSC colonies. This is why in the main text we checked for each single colony that the threshold time t_c that we are using to classify cells as G_0 is very distant not simply from the mean, but also from the bulk of the distribution of the division times of that colony.

Despite this separation, one could argue that the the G_0 criterion still influences some of our results. Indeed, a possible (wrong) explanation of the correlation between kinetics of the active cells and the frequency of G_0 cells would be the following: if a colony is slow, the divisions times of its cells are on average large, so that once in a while a cell may divide after an exceedingly long time, larger than the threshold t_c that we use to tag cells as G_0. Hence, the G_0 tag would not correspond to a *qualitatively* different cell state, but just to a *quantitative* epiphenomenon of having chosen an arbitrary threshold t_c. Were this the case, the correlation between the number of inactive cells (which is primarily determined by the G_0 cells) and the division time (which is determined by the active cells) would be trivial —in fact, even tautological— as both quantities would derive from the *same* actual probability distribution of division times, which we would be artificially separating into two parts: the active cells, $\tau < t_c$, and the inactive cells, $\tau > t_c$. This argument, however, is not consistent with the data, as we now show at a quantitative level.

Given a certain colony, we can use the gap-exponential probability distribution $P(\tau)$ fitted to the actual experimental histogram of the division times of that colony (Fig. 3a), and calculate the probability to find a cell that has a division time larger than t_c (i.e. a cell that would therefore be erroneously tagged as G_0 in our experiment, according to the argument above). This probability is given by,

$$P_{G_0} = e^{-\gamma(t_c - \tau_{\min})} \ . \tag{1}$$

Using the actual experimental value $t_c = 84$ h and the fitted values of γ and $\tau_{\min}$ in each one of our colonies, we find that this probability is always extremely small, ranging between 10^{-17} to 10^{-2}. This means that if in each colony we extracted the cell division times using its own probability and tagged as G_0 all cells with $\tau > t_c$, we would obtain a number of G_0 cells approximately equal to $P_{G_0} \times 128$, which yields *zero* in 29 out of 32 colonies, in stark contrast with their actual total number of G_0 cells, which is 310; in fact, even in the three colonies in which $P_{G_0} \times 128$ is non-zero, we would obtain 7, 2 and 1 G_0 cells, instead of the actual 22, 18, and 22 G_0 cells that those colonies actually have. We conclude that cells classified as G_0 in our experiments are *not* merely the very slow cells; the inactive cells sub-population is thus a *bona fide* qualitatively different group from that of the active cells. Therefore, the tautological explanation of the correlation between topology and kinetics does not stand.

Having established that the experimental value $t_c = 84$h allows unambiguous classification of inactive cells, we now explain the rationale for this specific choice. Ideally, a presumed G0 cell should be followed for as long as possible, within experimental constraints. In our case, the primary limitation is crowding. As proliferation proceeds, colony density progressively increases, so cells in later generations are born and migrate in an increasingly crowded environment. If a G0 cell appears at generation $k = 6$, after 84 hours all active $k = 6$ cells have divided—often more than once. At this stage, cells can be very close to one another and even overlap in three dimensions, making cell identification difficult (see middle panels in Fig. S1). Under these conditions, tracking individual cells becomes highly demanding, particularly because cell tracking is performed manually rather than automatically to ensure accurate identification. Extending the threshold beyond this value therefore appears impractical. Conversely, based on the considerations above, 84 hours already represents a robust criterion for classifying inactive cells. For comparison, previous studies on BMSC [1] used a shorter threshold of three days (72 hours) to define non-dividing cells.

A caveat on the sign of the correlation between topology and kinetics

It is important to note that the *positive* correlation between the number of inactive cells and the mean division time (Fig.5 of the main text) can only hold for colonies large enough to have some cells at generation $k = 7$ (as those included in this study); if, on the contrary, we selected very small colonies where *all* cells become G_0 early on in the development, so that no cells arrive at the seventh generation, we would enter a qualitatively different regime where N_{inactive} would be *proportional* to the size of the colony and where N_{missing} would always be equal to 128. If one is interested in that regime, other metrics should be used to describe the topology of the tree.

Supplementary equations

Exponential and gap-exponential distributions of division times

In simple models of cell proliferation in which division events occur at a constant rate γ, the increment dN of the number of cells N present at time t is proportional to the number itself, $dN = \gamma N dt$, whose mathematical solution is, $N(t) = N_0\, e^{\gamma t}$, which is the standard exponential proliferation (with N_0 cells at time zero); conversely, the number M of cells that *have not yet divided* at time t *decreases* with time, with a decrement given by $dM = -\gamma M dt$, so that $M(t) = N_0\, e^{-\gamma t}$, an exponential decay of the number of undivided cells. The probability $P(\tau)d\tau$ that the division time has a value between τ and $\tau + d\tau$, is equal to the number of cells not yet divided at time τ *minus* the number of cells not yet divided at time $\tau + d\tau$, normalized by the total number of initial cells, that is,

$$P(\tau)d\tau = \frac{M(\tau) - M(\tau + d\tau)}{N_0} = \frac{N_0\, e^{-\gamma\tau} - N_0\, e^{-\gamma(\tau+d\tau)}}{N_0} = e^{-\gamma\tau} - e^{-\gamma\tau}e^{-\gamma d\tau} = e^{-\gamma\tau} - e^{-\gamma\tau}(1 - \gamma d\tau) = e^{-\gamma\tau}\,\gamma d\tau\ , \quad (2)$$

where we have used Taylor's expansion of $e^{-\gamma d\tau} \approx (1 - \gamma d\tau)$ for small $d\tau$. We conclude that the probability density of the division time in the case of a proliferation process with constant rate γ is $P(\tau) = \gamma e^{-\gamma\tau}$. Notice that the rate γ has units of the inverse of a time, and the factor γ in front of the exponential is required to have a normalized probability, $\int d\tau\, P(\tau) = 1$. For this distribution, the mean division time is,

$$\langle\tau\rangle = \int_0^\infty d\tau\ \tau\, P(\tau) = \int_0^\infty d\tau\ \tau\, \gamma\, e^{-\gamma\tau} = \frac{1}{\gamma}\ . \quad (3)$$

On the other hand, if the distribution of the division times is a gap-exponential,

$$P(\tau) = \gamma\, \theta(\tau - \tau_{\min})\, e^{-\gamma(\tau-\tau_{\min})}\ , \quad (4)$$

the mean division time is given by,

$$\langle\tau\rangle = \int_0^\infty d\tau\ \tau\, P(\tau) = \int_{\tau_{\min}}^\infty d\tau\ \tau\, \gamma\, e^{-\gamma(\tau-\tau_{\min})} = \tau_{\min} + \frac{1}{\gamma}\ . \quad (5)$$

Synchronization vs correlation

To illustrate the mathematical mechanism behind synchronization, we analyze explicitly the case of sister cells. Consider a mother cell that divides at an absolute time t_m, which is also the birth time of its two daughters. If we call τ_{s1} and τ_{s2} the division times of the two sisters, sister 1 will divide at a time $t_{s1} = t_m + \tau_{s1}$ and sister 2 at $t_{s2} = t_m + \tau_{s2}$; perfect synchronization of the daughters occurs when $t_{s1} = t_{s2}$. The square *desync* is the square difference of the absolute division times of the two sister cells, $(\Delta t)^2 = (t_{s1} - t_{s2})^2$. We can average the square desync over all divisions within the same generation, a quantity that we will indicate as $\langle(\Delta t)^2\rangle$. By using the definition of t_{s1} and t_{s2} given above, we obtain,

$$\left\langle(\Delta t)^2\right\rangle = \left\langle(t_{s1} - t_{s2})^2\right\rangle = \left\langle(t_m + \tau_{s1} - t_m - \tau_{s2})^2\right\rangle = \left\langle\tau_{s1}^2\right\rangle + \left\langle\tau_{s2}^2\right\rangle - 2\left\langle\tau_{s1}\tau_{s2}\right\rangle\ . \quad (6)$$

Once we average over all events, all single cell means will be the same, hence $\left\langle\tau_{s1}^2\right\rangle = \left\langle\tau_{s2}^2\right\rangle = \left\langle\tau^2\right\rangle$. In absence of correlation we have, $\langle\tau_{s1}\tau_{s2}\rangle = \langle\tau_{s1}\rangle\langle\tau_{s2}\rangle = \langle\tau\rangle^2$. In this way the square desync becomes,

$$\left\langle(\Delta t)^2\right\rangle = 2\left(\left\langle\tau^2\right\rangle - \langle\tau\rangle^2\right)\ , \quad (7)$$

which is just twice the variance of the distribution of the division times, σ_τ^2; hence the desync in absence of correlation is given by,

$$\left\langle(\Delta t)^2\right\rangle = 2\sigma_\tau^2\ . \quad (8)$$

Instead, if we do not assume that the sisters' division times are uncorrelated, we can rewrite the equation (6) in the following way,

$$\left\langle(\Delta t)^2\right\rangle = \left\langle\tau_{s1}^2\right\rangle + \left\langle\tau_{s2}^2\right\rangle - 2\langle\tau_{s1}\rangle\langle\tau_{s2}\rangle + 2\langle\tau_{s1}\rangle\langle\tau_{s2}\rangle - 2\langle\tau_{s1}\tau_{s2}\rangle = 2\left(\left\langle\tau^2\right\rangle - \langle\tau\rangle^2\right) - 2\left(\langle\tau_{s1}\tau_{s2}\rangle - \langle\tau_{s1}\rangle\langle\tau_{s2}\rangle\right)\ , \quad (9)$$

where we recall that $\left\langle\tau^2\right\rangle - \langle\tau\rangle^2 = \sigma_\tau^2$ is the variance of the division times. If we now introduce the Pearson correlation coefficient, ρ_{P}, between the division times,

$$\rho_{\mathrm{P}} = \frac{\langle\tau_{s1}\tau_{s2}\rangle - \langle\tau_{s1}\rangle\langle\tau_{s2}\rangle}{\sigma_\tau^2}\ , \quad (10)$$

we finally obtain that the square desync with correlated sisters' division times is given by,

$$\left\langle (\Delta t)^2 \right\rangle = 2\sigma_\tau^2 \left(1-\rho_{\mathrm{P}}\right) \ . \tag{11}$$

What originates the presence of correlations is, as mentioned in the main text, an open problem. We have statistical indicators suggesting that density and cell-cell contact do not have a major role in this respect. The reason is that - even if density and crowding effects increase as growth progresses - cell motility remains quite effective even at the largest time considered. From Fig. S2, we can see that active cells move approximately $6-10$ body-lengths during their cell cycle. Since movements are random in space, this means that sister cells are typically far apart from each other when they divide. Indeed, if we directly compute the median distance between sister cells at the end of their life cycle, we find $d = 0.43$ mm (i.e. of the order of 10 body-lengths). This means that sister cells are not in physical contact or proximal to one another when they divide.

The branching process model

Here we give the solution of the branching process used in the main text to model the growth of the number of cells in a colony. A branching process [12] is a stochastic process for the evolution of a population where one assumes [13, Ch. III] that (i) each individual (here, a cell) starts its own family of descendants; (ii) all the families have the same stochastic properties; (iii) the families do not interact with each other (no inter-marriage). At any given time one has $N(t)$ cells, whose life can end with some probability per unit time $\gamma(\tau)$, which in general depends on each individual's age (and is thus different for each), and in this sense the process is non-Markovian. At the end of its life, the individual is replaced by r other identical individuals with probability p_r; here we allow only $r = 0,2$ (i.e. apoptosis or cell division). Alternatively, a Markovian description can be obtained if the state of the population is described not simply by the number of objects present but by a list of objects with an age and a lifetime [12, Ch. VI].

One defines the conditional probability $P(N,t|m,0)$ of having N cells in the colony at time t given that there were m cells at the beginning ($t = 0$). A consequence of the fact that different families do not interact is that we can write $P(N,t|m,0)$ as the convolution of m factors $P(N,t|1,0)$,

$$P(N,t|m,0) = \sum_{s_1=0}^{N} \ldots \sum_{s_{m-1}=0}^{N-s_1-\ldots-s_{m-2}} P(N-s_1-\ldots-s_{m-1},t|1,0)P(s_1,t|1,0)\ldots P(s_{m-1},t|1,0) \tag{12}$$

Now we introduce the generating function [12, 13],

$$F(z,t|m,0) = \sum_{n=0}^{\infty} z^n P(n,t|m,0), \tag{13}$$

with $t \geq 0$ and $|z| \leq 1$. It can be shown that the generating function of a convolution is the product of the generating functions [13], so that

$$F(z,t|m,0) = [F(z,t|1,0)]^m. \tag{14}$$

We can then focus on $P_n(t) = P(n,t|1,0)$ and its generating function, which we write simply as $g(z,t) = F(z,t|1,0)$.

The probability that a cell of age τ ends its life (through either division or apoptosis) in the next infinitesimal time interval $d\tau$ is $\gamma(\tau)d\tau$. Calling $w(\tau)$ the probability for the cell to reach age τ without death or division, it holds that

$$dw(\tau) = -\gamma(\tau)w(\tau)d\tau \tag{15}$$

(note that this is different from the probability to divide or die exactly at age τ, defined below). Solving the differential equation for $w(\tau)$,

$$w(t) = e^{-\int_0^t dt'\gamma(t')}, \tag{16}$$

and the probability to reach age τ and undergoing an event between τ and $\tau + d\tau$ is

$$p(\tau)\,d\tau = w(\tau)\gamma(\tau)\,d\tau = -\frac{dw(\tau)}{d\tau}\,d\tau. \tag{17}$$

With these definitions, an integral equation for the generating function can be found [12]:

$$g(z,t) = zw(t) - \int_0^t h[g(z,t-t')]\,dw(t'), \tag{18}$$

where $t \geq 0$, $|z| \leq 1$, $h(s)$ is the generating function of the probability for the number of descendants,

$$h(s) = \sum_{r=0}^{\infty} p_r s^r. \tag{19}$$

Following the procedure used in [14] we finally obtain an equation for the average number of cells as function of absolute time:

$$\langle N(t) \rangle - 1 = \int_0^t [m\langle N(\tau)\rangle - 1]\gamma(t-\tau)w(t-\tau)\,d\tau, \tag{20}$$

where $m = h'(s)|_{s=1}$. The solution $\langle N(t)\rangle$ can be shown to be unique and bounded in every finite interval $[0,t]$.

Equation (20) is quite general; to attempt to solve it, explicit expressions for $\gamma(\tau)$ and p_r must be given. For the rate $\gamma(\tau)$ we choose

$$\gamma(\tau) = \Theta(\tau - \tau_{\min})\gamma, \tag{21}$$

i.e. zero probability for $\tau \leq \tau_{\min}$, and a constant rate for larger ages. Through (16) and (17), this leads to the gap-exponential distribution of cell cycle duration used in the main text,

$$p(t) = \gamma\Theta(t - \tau_{\min})e^{-\gamma(t-\tau_{\min})}. \tag{22}$$

For the probability of descendants we choose

$$p_r = p_0\delta_{r,0} + (1-p_0)\delta_{r,2}, \tag{23}$$

i.e. the cell can either die or divide (hence p_0 is the probability of apoptosis). In this model, then, apoptosis only occurs at the end of the cell cycle. Given that apoptosis is more likely to occur after the end of the G_1 phase [15] (which is typically the longest), and that apoptosis occurs with low probability in our experiments, this limitation should not be problematic.

We still need to add the probability of entering the G_0 phase. We do this by modifying $p(t)$: the probability of undergoing division or death in the interval $[t_1,t_2]$ is $\int_{t_1}^{t_2} p(t)\,dt$. Expression (22) is normalized such that $\int_0^\infty p(t)\,dt = 1$, i.e. all cells die or divide. The most convenient way to introduce G_0 is not through a new transition but simply allowing some probability for a cell to never finish the cycle: we thus repalace (22) by

$$p(t) = a\gamma\Theta(t-\tau_{\min})e^{-\gamma(t-\tau_{\min})}\ , \tag{24}$$

with $a < 1$, so that $1-a$ is the probability to never divide or die, i.e. to become G_0 (see [16] for a different approach).

Now the equation for $\langle N(t)\rangle$ can be solved using Laplace transforms, adapting the procedure of [14] for the present case with two additional parameters. Introducing the Laplace transforms

$$n(s) = \int_0^\infty e^{-st}\langle N(t)\rangle\,dt, \tag{25}$$

$$y(s) = \int_0^\infty e^{-st}w(t)\gamma(t)dt = \int_0^\infty e^{-st}p(t)\,dt = a\cdot\frac{\gamma}{s+\gamma}e^{-s\tau_{\min}}, \tag{26}$$

where $s \in \mathbb{C}$, equation (20) is solved in the Laplace domain as

$$n(s) = \frac{1}{s}\frac{1-y(s)}{1-m\cdot y(s)}, \tag{27}$$

and, going back to the time domain,

$$\langle N(t)\rangle = \frac{1}{2\pi i}\int_{\alpha-i\infty}^{\alpha+i\infty}\left[\frac{1}{s} + \frac{\gamma(m-1)a\cdot e^{-s\tau_{\min}}}{s\phi(s)}\right]e^{st}\,ds, \tag{28}$$

where $\phi(s) = s + \gamma(1 - ma\cdot e^{-s\tau_{\min}})$ and α is a real number such that the integration contour lies to the right of all the poles of the integrand. The integration is done using the residue theorem; the integrand has poles at $\hat{s} = 0$ and at

$$\hat{s}_k = -\gamma + \frac{1}{\tau_{\min}}W_k(m\gamma a\tau_{\min}e^{\gamma\tau_{\min}}), \tag{29}$$

where W_k is the k-*th* branch of the Lambert, or product log, function. The residue theorem gives

$$\langle N(t)\rangle = 1 + \Theta(t-\tau_{\min})\cdot\left[-\frac{(m-1)a}{(ma-1)} + \sum_{k=-\infty}^{+\infty}\gamma(m-1)a\frac{e^{\hat{s}_k(t-\tau_{\min})}}{\hat{s}_k[1+\tau_{\min}(\hat{s}_k+\gamma)]}\right]. \tag{30}$$

Finally, from (23) and (19) we have $h(s) = p_0 + (1 - p_0)s^2$, so that

$$m = h'(s)|_{s=1} = 2(1 - p_0), \tag{31}$$

and therefore

$$\langle N(t) \rangle = n_0 + n_0 \cdot \Theta(t - \tau_{\min}) \left[-\frac{(1-2p_0)a}{2a(1-p_0)-1} + \sum_{k=-\infty}^{+\infty} \gamma(1-2p_0)a \frac{e^{\hat{s}_k(t-\tau_{\min})}}{\hat{s}_k[1+\tau_{\min}(\hat{s}_k+\gamma)]} \right] . \tag{32}$$

This is the function we use to predict and fit the experimental growth curves in the main text.
The red and green dashed curves shown in Fig. 6 are obtained by plugging the correct set of parameters (γ, $\tau_{\min}$, p_0, a) for each colony in the previous expression and computing $\langle N(t) \rangle$ accordingly. The four parameters used for the red dashed line were obtained directly from the experimental data: γ and $\tau_{\min}$ are obtained by fitting the experimental probability distribution of the division times $P(\tau)$, while p_0 and a are obtained by simply applying, for each colony,

$$p_0 = \frac{\text{\# dead cells}}{\text{\# dead cells + \# active cells}} \tag{33}$$

$$a = 1 - \frac{\text{\# } G_0 \text{ cells}}{\text{\# } G_0 \text{ cells + \# active cells + \# dead cells}} \tag{34}$$

On the other hand, the four parameters used for the green dashed line are obtained through a least square fit of equation (32) on the experimental $N(t)$ in the temporal range $[t_0, t_0 + 5\bar{\tau}]$, where t_0 is the absolute time at which the first cells of the colony undergoes mitosis and $\bar{\tau}$ is the average division time of the specific colony we are considering.

Supplementary table

experiment [1]	DT (min) [2]	donor [3]	age [4]	sex [5]	passage [6]	colony [7]	N_G0 [8]	N_dead [9]	N_inactive [10]	N_miss [11]	mean tau (hours) [12]	gamma (1/hours) [13]	tau_min (hours) [14]
20230113	20	5	35	M	0	01	6	0	6	12	14.83	0.302	11.29
						07	0	0	0	0	12.97	0.527	11.20
						08	6	1	7	16	19.54	0.242	14.68
						09	14	0	14	100	19.87	0.189	14.12
20230503	20	6	32	M	0	13	11	0	11	24	19.78	0.205	14.25
						16	12	2	14	60	23.15	0.138	14.92
20240627	15	7	65	F	2	03	12	0	12	32	20.44	0.122	11.71
						04	18	4	22	120	26.16	0.055	8.41
						06	22	0	22	120	35.45	0.04	12.48
20240924	15	7	65	F	1	05	22	0	22	102	21.40	0.104	11.98
						12	23	0	23	76	21.20	0.096	10.60
20241017	15	10	62	F	1	02	0	0	0	0	14.74	0.538	13.14
						05	17	3	20	80	20.61	0.127	10.93
						09	12	1	13	90	18.52	0.158	11.62
						11	10	0	10	26	21.00	0.128	12.06
						16	2	1	3	8	17.62	0.209	12.33
20241112	15	10	62	F	1	02	1	1	2	4	19.18	0.213	14.29
						05	1	0	1	4	15.82	0.328	12.73
20241203	15	10	62	F	1	03	6	0	6	60	18.67	0.202	13.93
						07	22	2	24	100	24.92	0.061	8.47
						09	5	0	5	22	20.19	0.117	11.00
20250313	15	9	45	M	2	03	0	0	0	0	18.33	0.167	12.32
						14	16	0	16	100	21.06	0.115	11.45
20250328	15	12	43	F	1	02	3	0	3	12	19.60	0.18	13.85
						06	0	2	2	8	18.17	0.331	15.39
						11	6	0	6	12	24.29	0.111	14.94
						12	19	0	19	96	24.88	0.095	13.73
						16	2	0	2	6	17.89	0.235	13.77
20250612	15	12	43	F	1	7	5	0	5	18	20.25	0.247	15.81
						9	30	0	30	76	24.69	0.11	16.06
20250626	15	12	43	F	3	3	7	0	7	16	22.78	0.157	15.46
						5	5	0	5	22	19.49	0.296	15.61

Table S1. Detailed parameters of all colonies. [1] Date of the experiment; [2] time interval between frames in the time lapse; [3] donor's label; [4] donor's age; [5] donor's sex; [6] passage; [7] label of the colony within that experiment; [8] number of G_0 cells, N_{G_0}; [9] number of dead cells, N_{dead}; [10] number of inactive cells, $N_{\mathrm{inactive}} = N_{G_0} + N_{\mathrm{dead}}$; [11] number of missing cells at generation $k = 7$, N_{missing}; [12] mean division time of the colony, $\bar{\tau}$; [13] proliferation rate, γ, obtained from the gap-exponential fit; [14] minimum division time, τ_{min}, obtained from the gap-exponential fit.

Supplementary videos

Video S1. Development of a colony starting from a single cell. Time-lapse video showing the development of colony `20250328_16`, starting from a single cell and progressing until confluence is reached. This colony was selected as it exhibits moderate growth and is representative of the average behaviour observed in the colonies analysed in this study. The video spans slightly more than 10 days and 14 hours of real time, with each second in the video corresponding to 6 hours. The timer displayed in the bottom right corner begins at 00d:00h:00m, corresponding to the frame in which the initial cell undergoes mitosis to give birth to two daughter cells. As the colony expands and cells move across the field of view, the video zooms out to ensure all cells remain visible. These changes in magnification can be tracked using the scale bar in the bottom left corner. A colour shift in the time from white to yellow, occurring between days 5 and 6, marks the final frame included in the quantitative analysis. At this point, all cells had either become inactive or reached the seventh generation.

Video S2. Synchronization of cells' mitosis. Time-lapse video showing the near-perfect synchronization of the mitosis of the four cousin cells of generation $k = 2$. Frames are separated by 15 minutes intervals.

Supplementary Figures

Figure S1

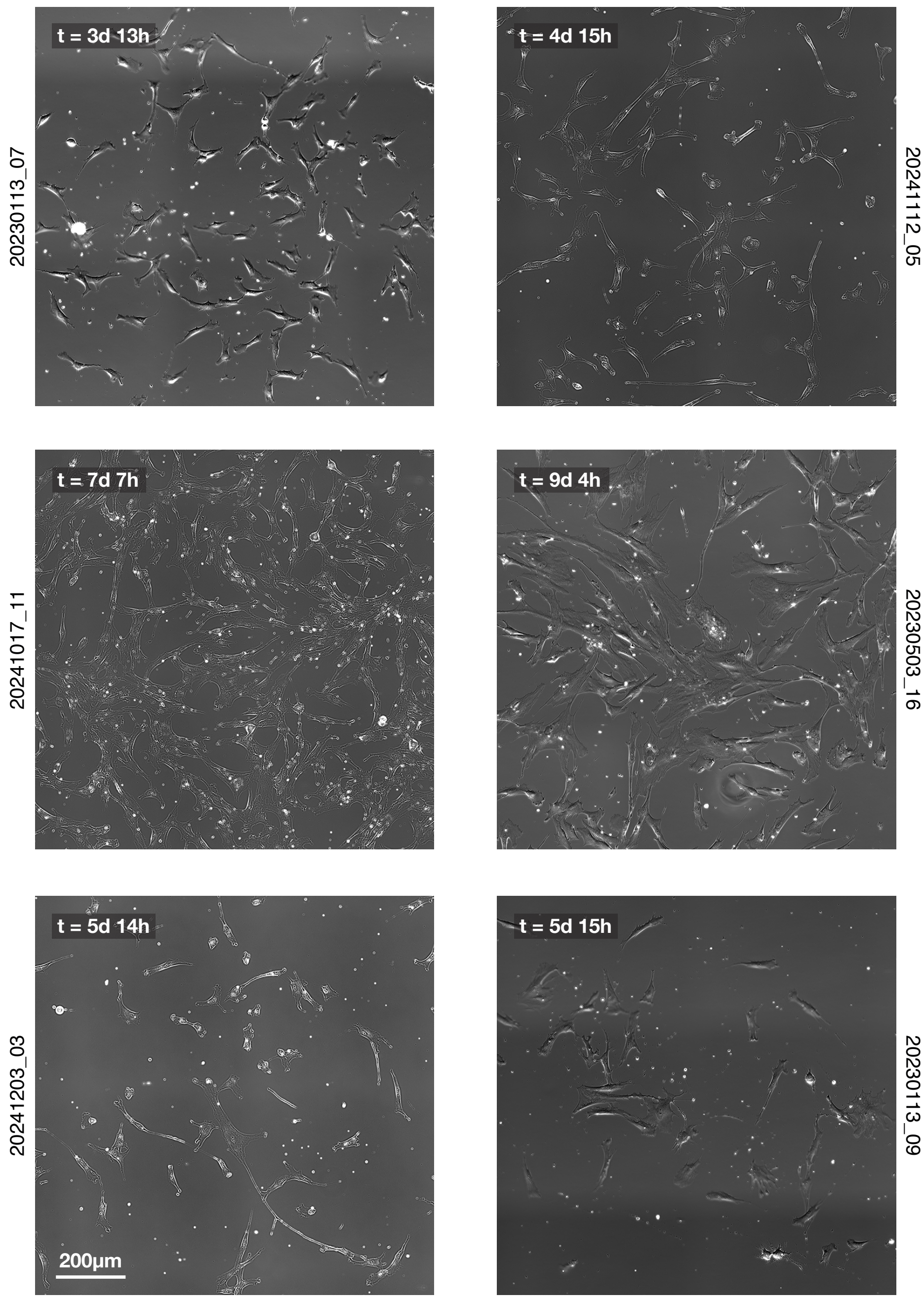

Figure S1. Examples of the colonies imaging data. Sample crops ($1.14mm \times 1.14mm$) of the images, at 20x magnification, for the colonies shown in Fig.2 and Fig.3 of the main text. All images are shown at the same spatial scale, and at the time of the last division of the sixth generation. In each colony, the time is calculated from the first mitosis, which defines the time origin (t = 0). Colony growth results in cells becoming progressively closer to one another and frequently overlapping, making cell identification difficult and leads to a substantial increase in the effort required for tracking beyond the seventh generation.

Figure S2

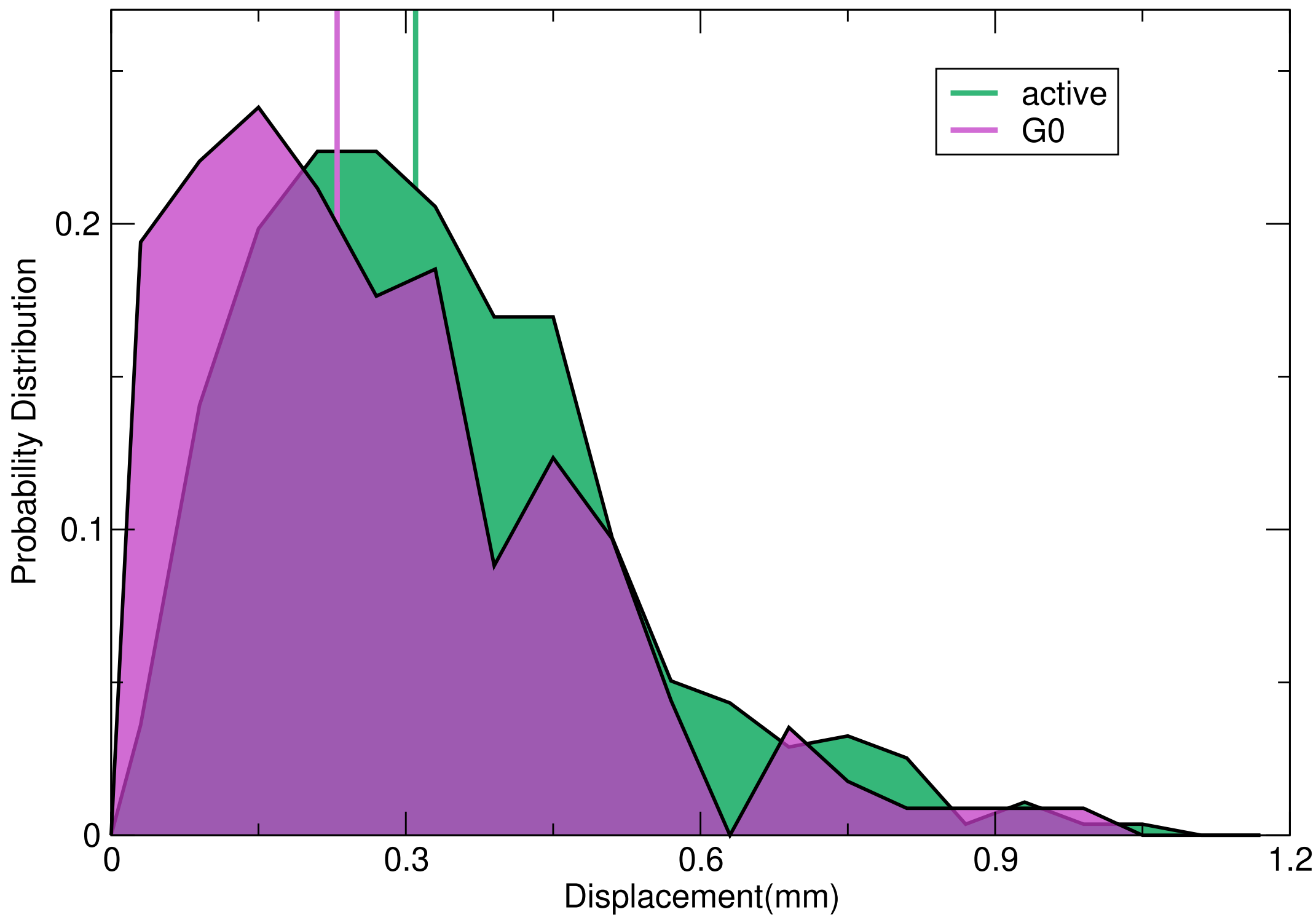

Figure S2. Motility of active cells vs G_0 cells. We considered the nine colonies with more than 15 inactive cells. For each active cell (a total of 462), we computed the spatial displacement between the position where it was born and the position where its division takes place. For inactive cells (a total of 189), for which division does not occur, we considered the division time of the colony, τ, based on all the active cells, and we computed the spatial displacement between the cell position 84 hours after birth and the position occupied τ hours earlier. The probability distributions of these displacements - pink for inactive cells and green for active cells - indicate that inactive cells tend to have a reduced motility, with a median displacement of 0.23mm (highlighted with the vertical pink line), against the median 0.31mm of active cells (vertical green line). However, the high overlap between the two histograms indicates that a large part of inactive cells have a motility comparable with the active ones. Therefore, at least in the first stages of the colony growth, we cannot exclude that, by maintaining morphology and mobility characteristics of active cells, inactive cells might retain robust stem cell potential. It is important to stress, though, that the definition of displacement we present here gives a lower bound on the actual distance traveled by individual cells, since a cell may first move away from its birth position and then return nearby. This underestimation is more severe in cells that present high motility, i.e. active cells. Consequently, we expect the difference in motility between the two populations to be larger when quantified in terms of the actual total distance traveled.

Figure S3

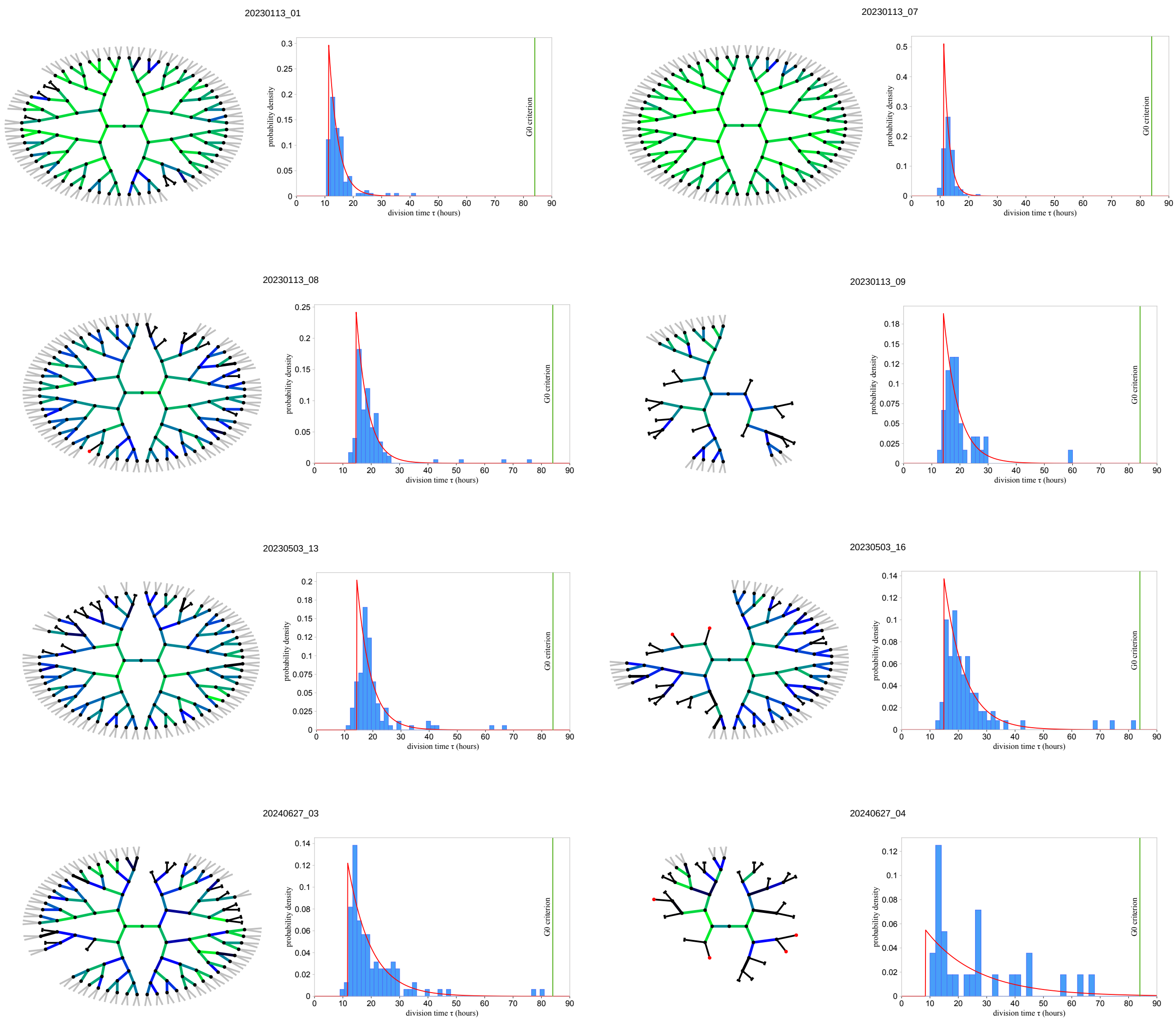

Figure S3. Lineage trees and division times histograms of all colonies. Abstract representation of the lineages of all the BMSC colonies in our dataset, including those reported in Fig. 2. For each colony we also report the corresponding normalised histogram of the cells' division times, analogous to the ones in Fig. 3.

Figure S3 (continued)

20240627_06

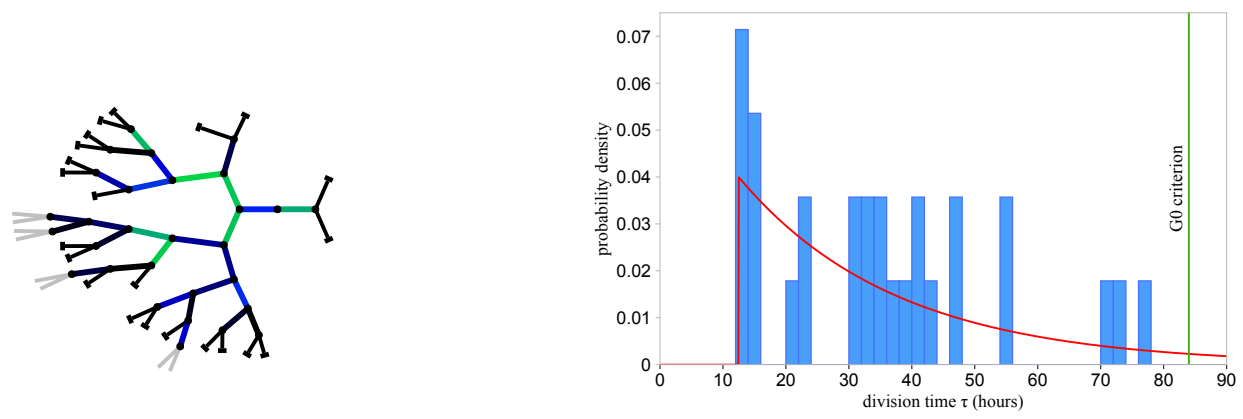

20240924_05

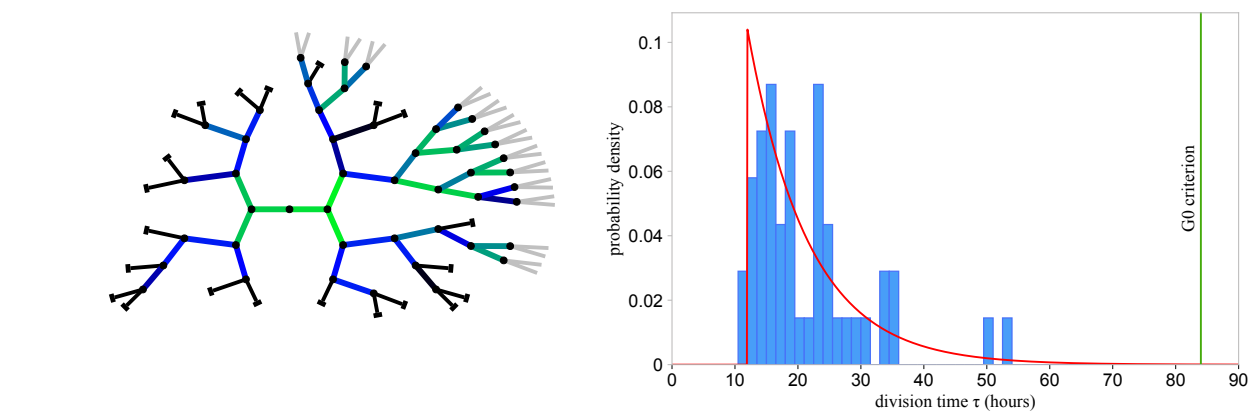

20240924_12

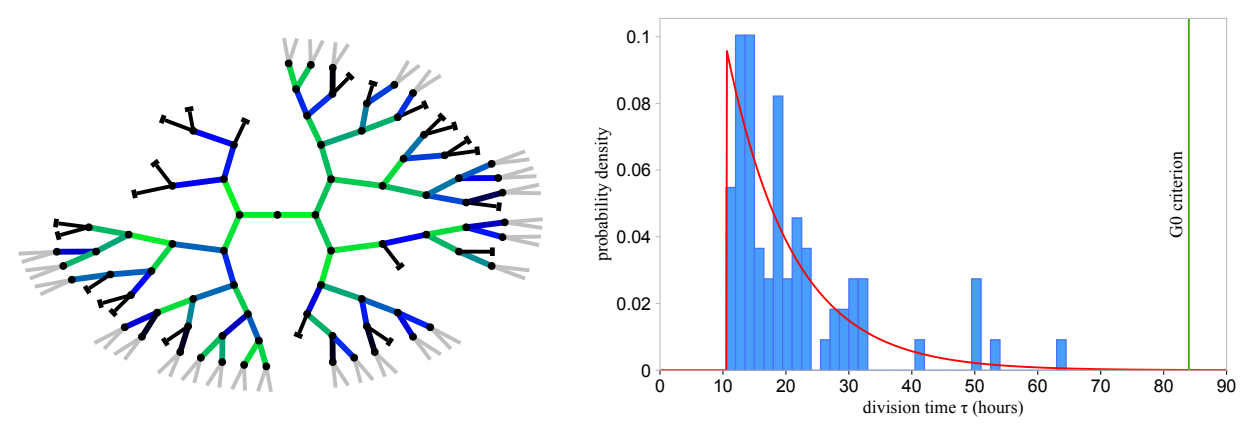

20241017_02

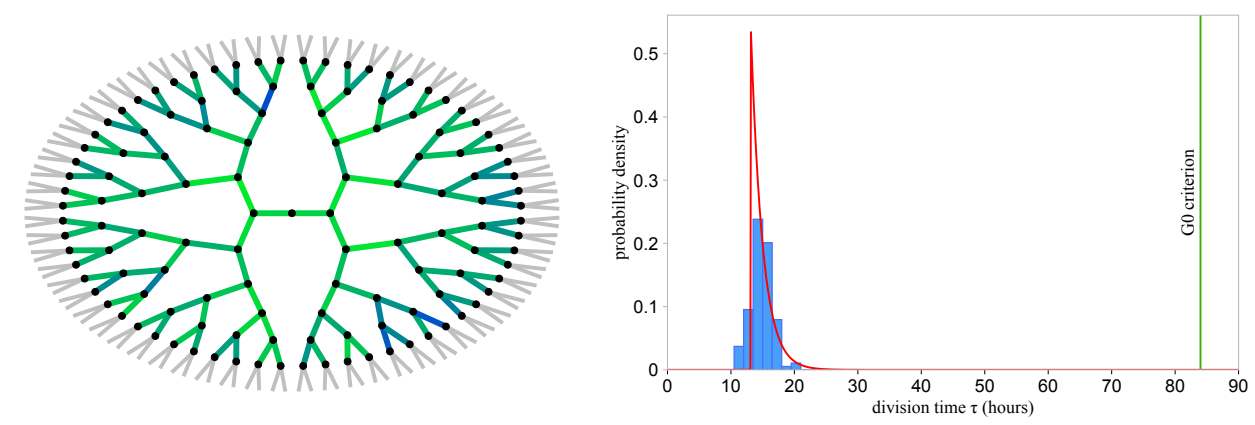

20241017_05

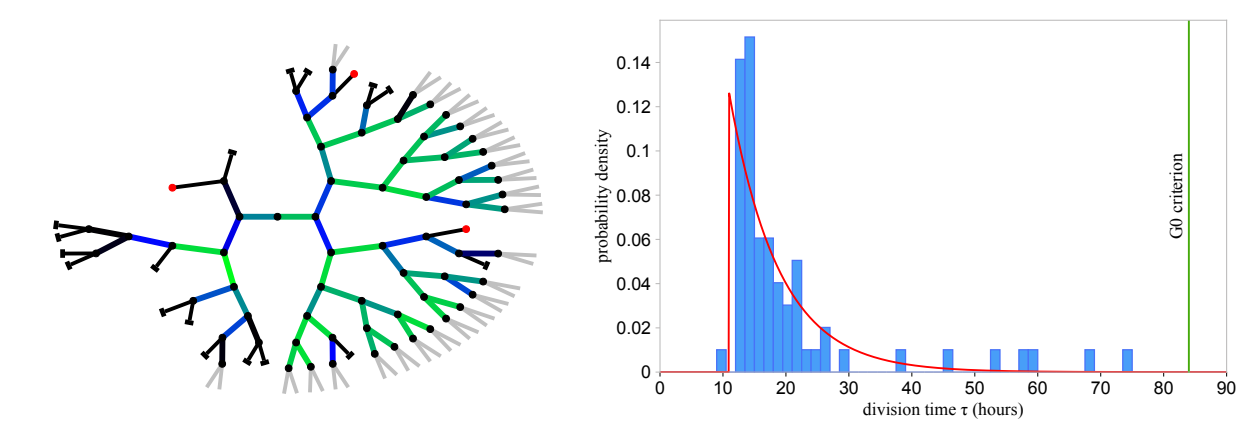

20241017_09

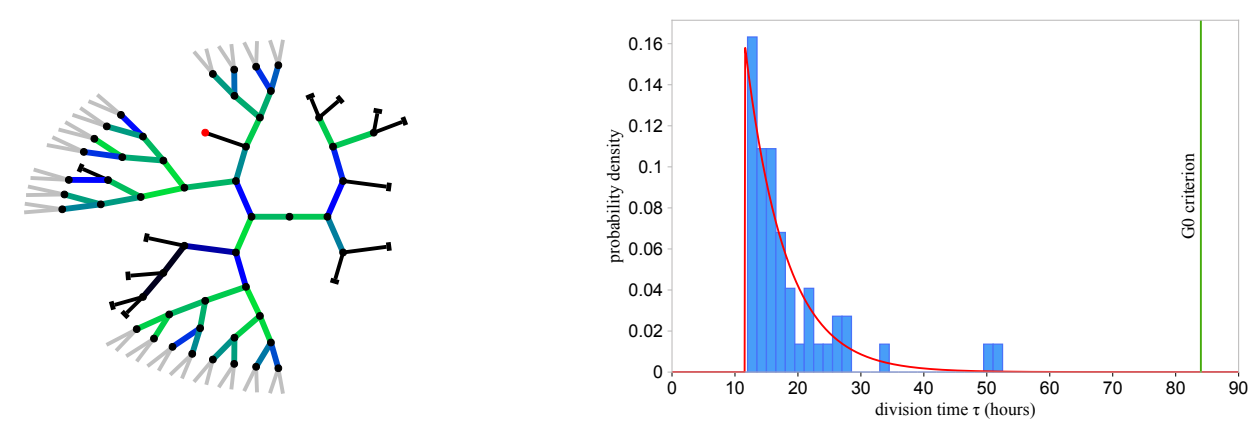

20241017_11

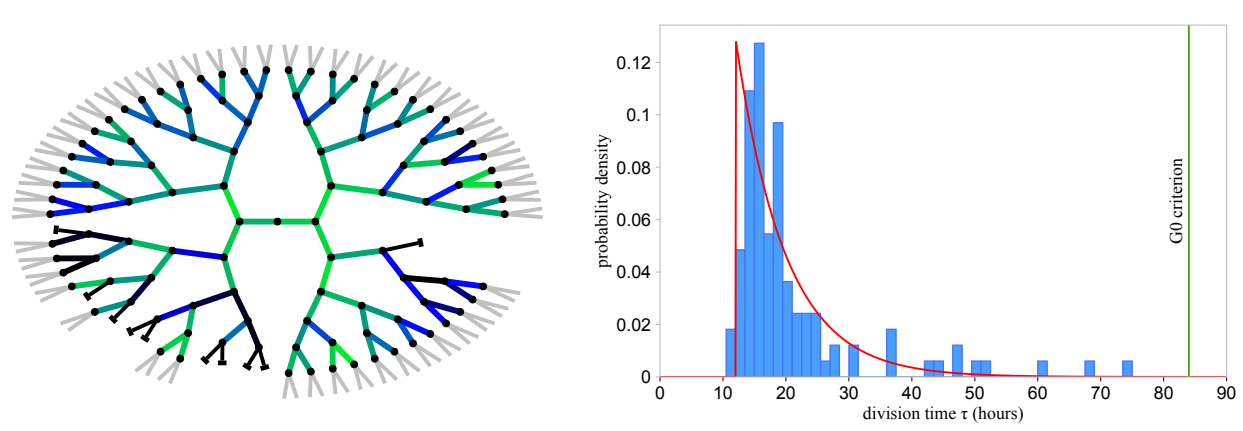

20241017_16

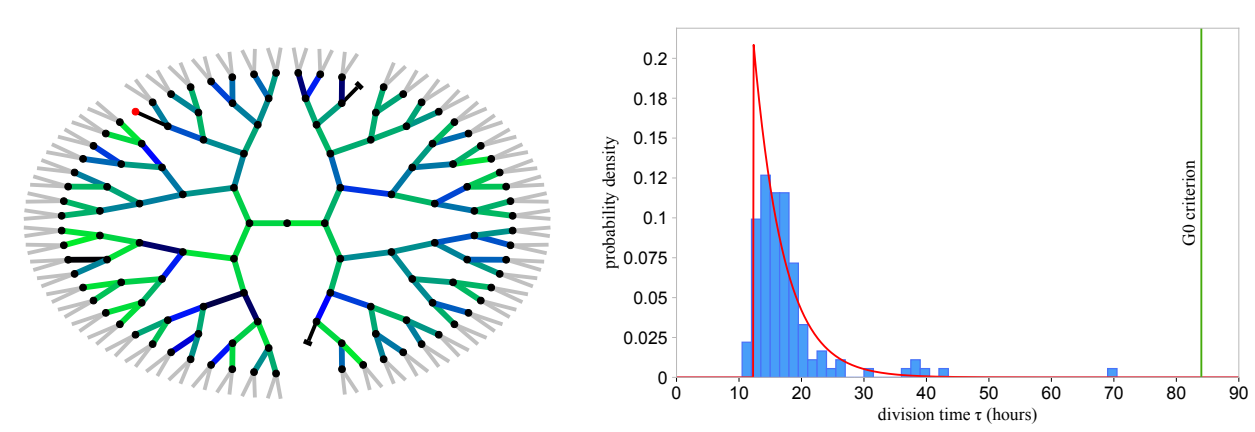

20241112_02

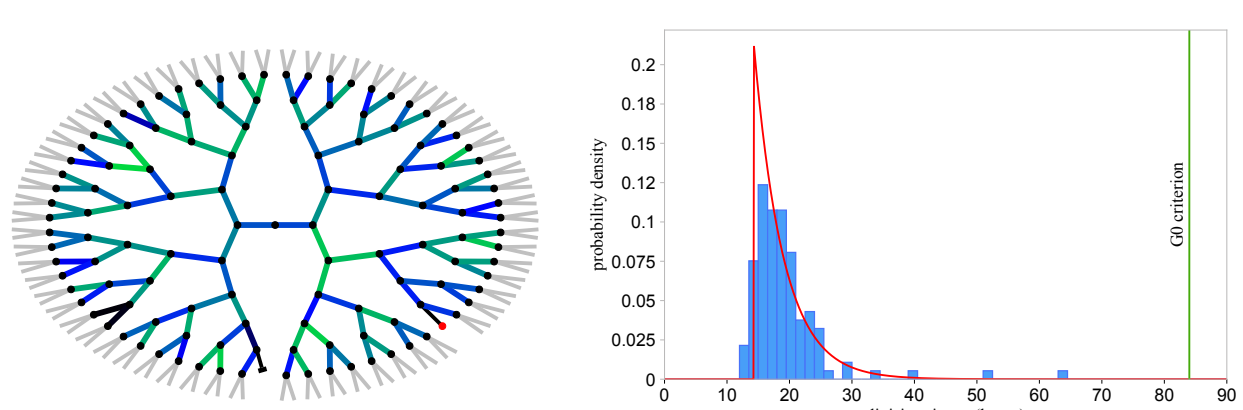

20241112_05

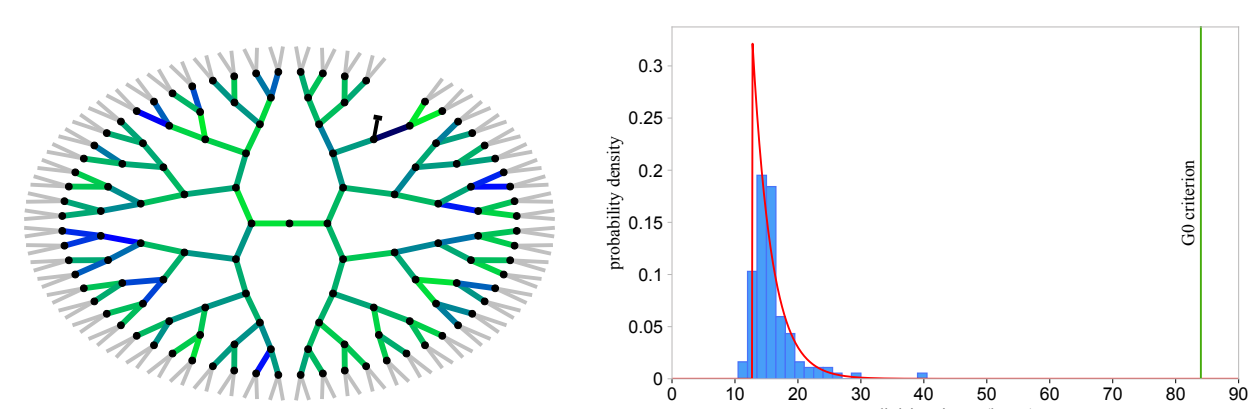

20241203_03

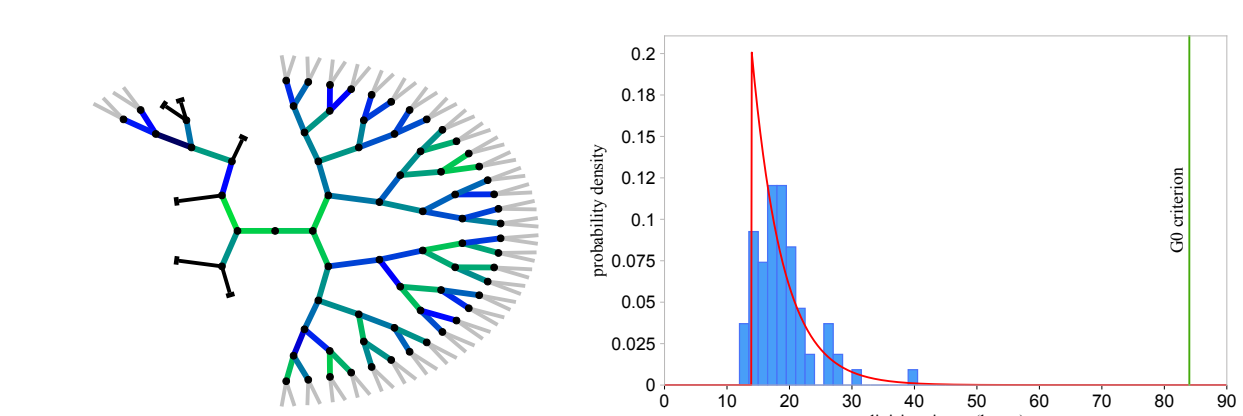

20241203_07

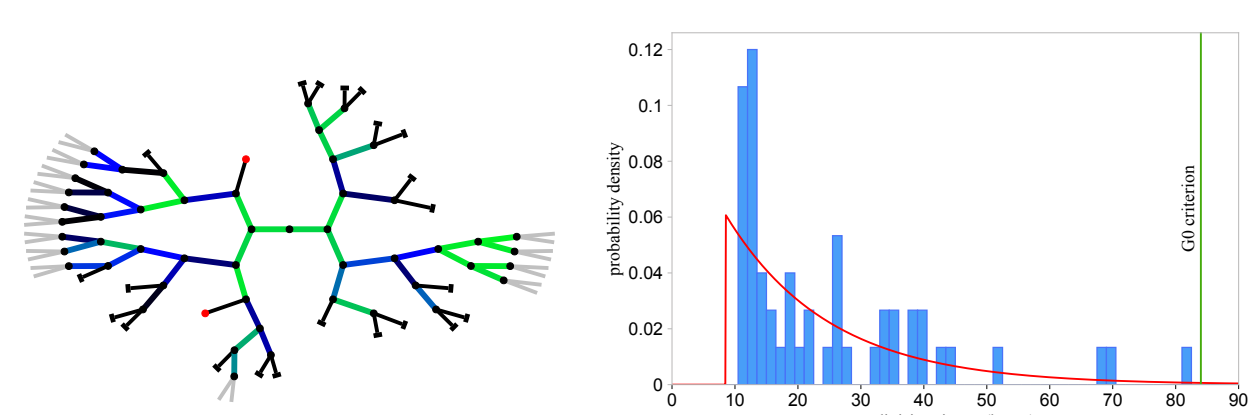

Figure S3 (continued)

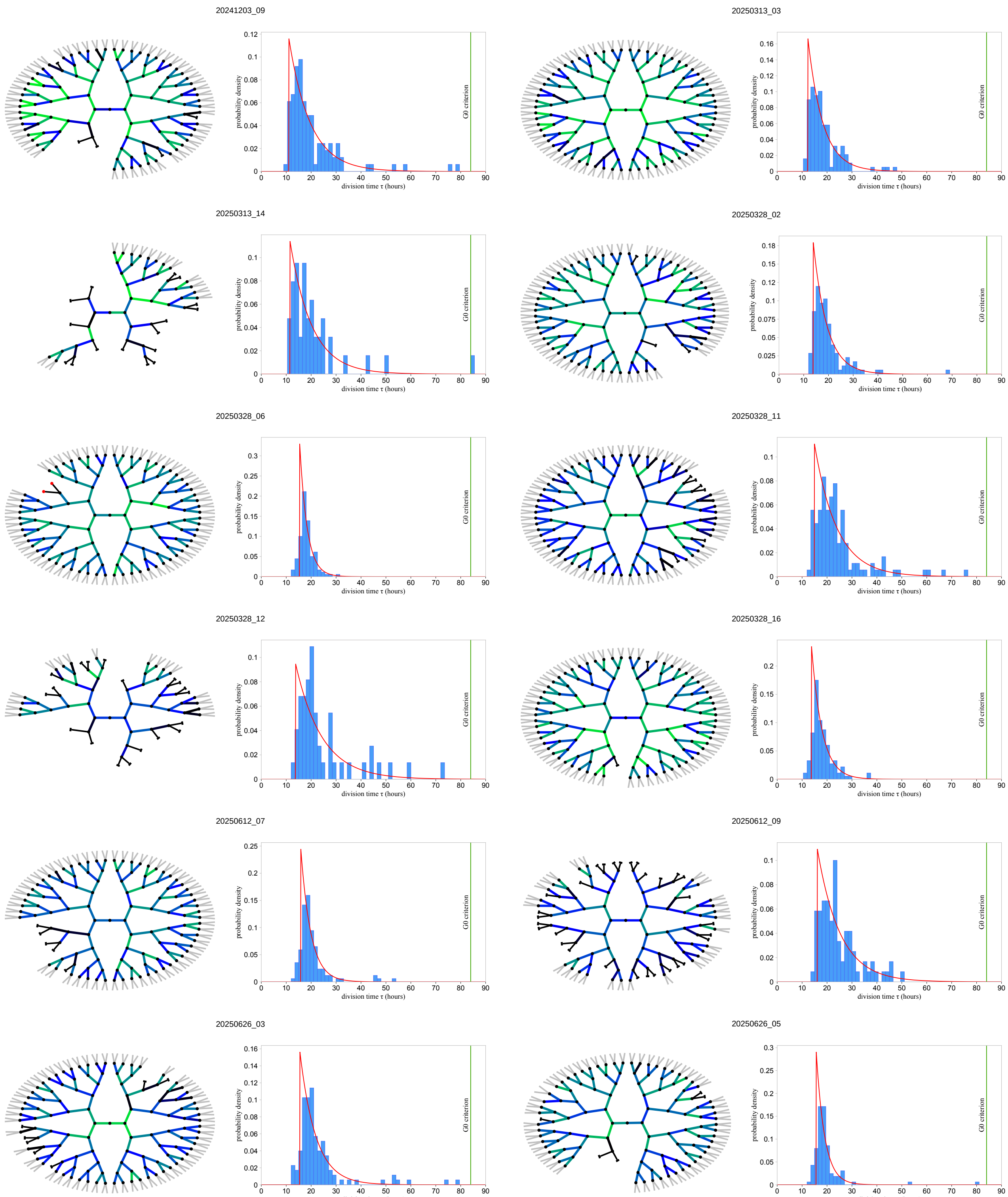

Figure S4

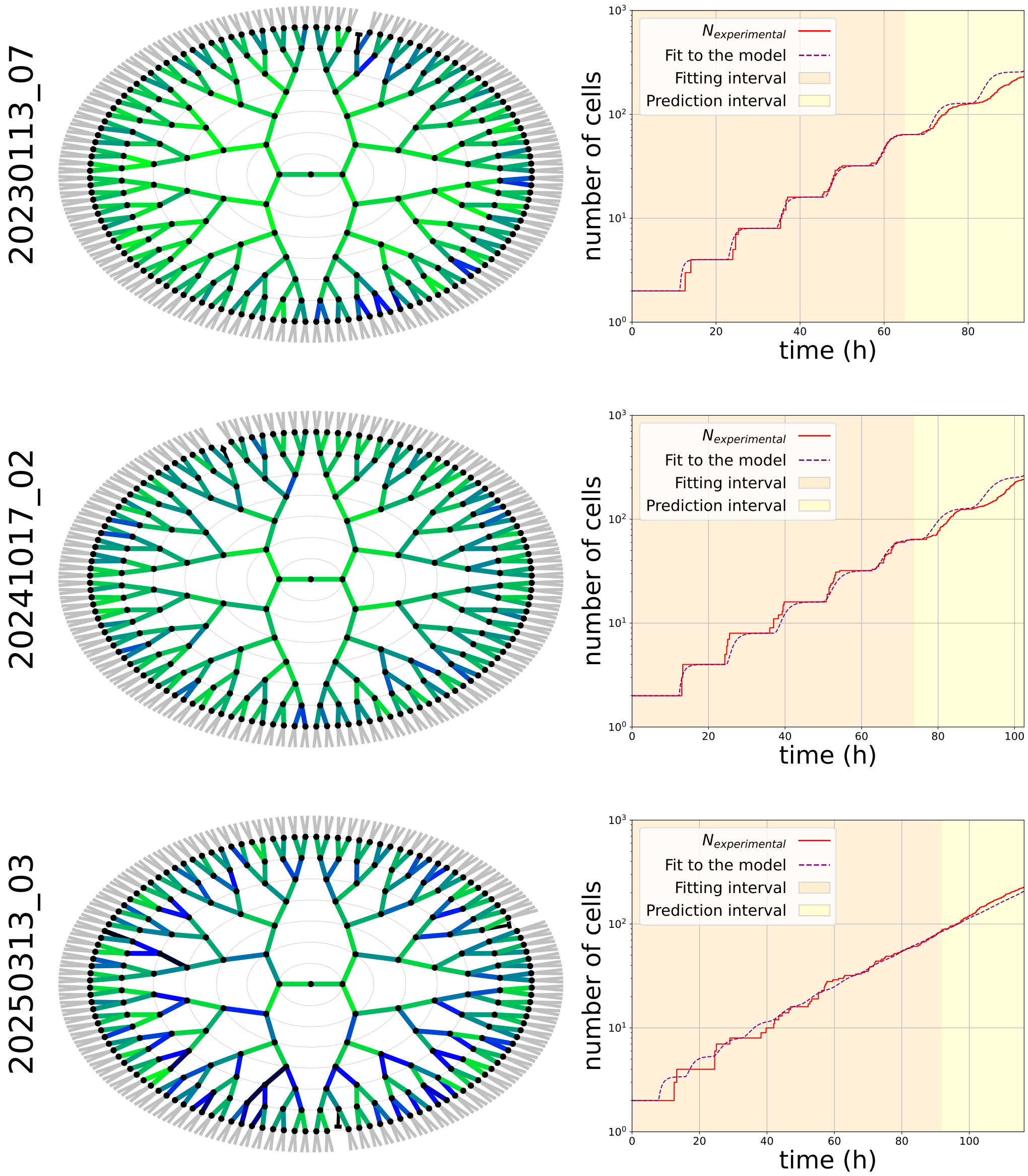

Figure S4: Predictive capacity of the branching process model. In the three colonies that have zero inactive cells up to generation 7, we push the tracking up to generation 8 (lineages on the left). We then employ the branching process model, with the parameters fitted on the previous 7 generations (orange interval), to see how good is the model's prediction on the total number of cells, $N(t)$, for the new 8th generation (yellow interval). For the two colonies with the strongest synchronization (sharpest steps - top panels) the prediction of the model in the new tracked region is not excellent; the actual number of cells seems to indicate that the real colony is slowing down compared to the predicted growth rate of the model. On the other hand, in the one colony with weak synchronization (bottom panels), the model's prediction is fairly accurate. We know that synchronization is made stronger by the presence of correlations between the division times of same-generation kin cells (sister-sister, cousin-cousin, etc), correlations that the branching process model does not include; hence, this result suggests that the failure of the model to predict the number of cells in colonies with the highest proliferative capacity is due to the existence of strong cell-cell correlations. (The black diamond at the last generation in the lineage of `20241017_02` indicates a cell whose fate is unknown as it crawls over the border of the plate, thus becoming out of focus.)

Figure S5

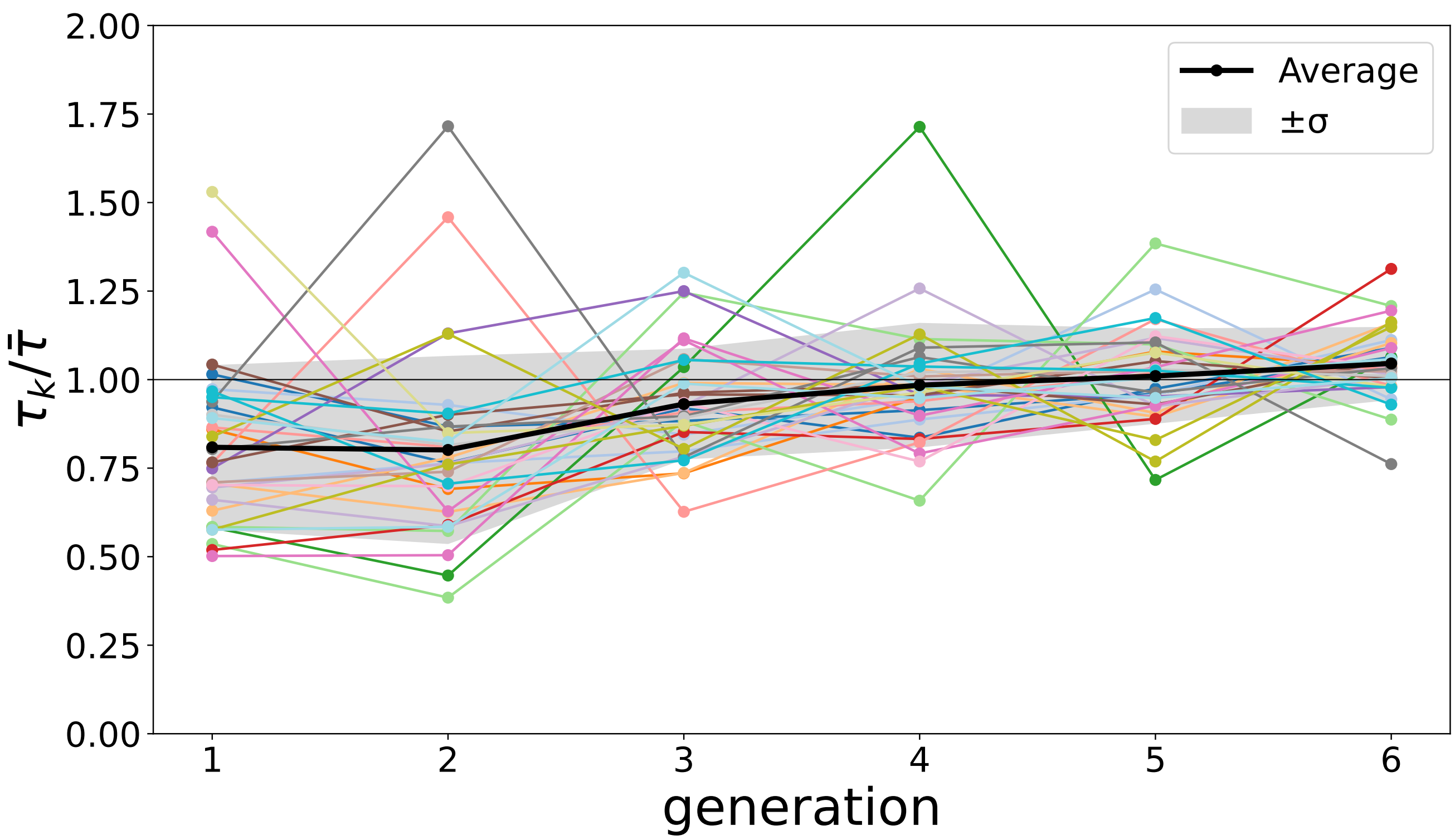

Figure S5: Average division time vs. generation. For each colony, the average division time at fixed generation, τ_k, divided by the colony's global average division time, $\overline{\tau}$, is plotted against generation number, k. The black curve is the average of $\tau_k/\overline{\tau}$ over all colonies. Despite fluctuations, a global trend toward increasing division times with generation is observed. Some colonies show a dip in division time at generation $k = 2$, but this is not detectable in the global average (the drop between $k = 1$ and $k = 2$ observed in some of the colonies may be ascribed to trypsinisation [17]). Notice that later generations have more cells than earlier ones, hence they weight more in the determination of $\overline{\tau}$; this is the reason why the black curve has a smaller offset with respect to 1 in later generations than in earlier ones.

Figure S6

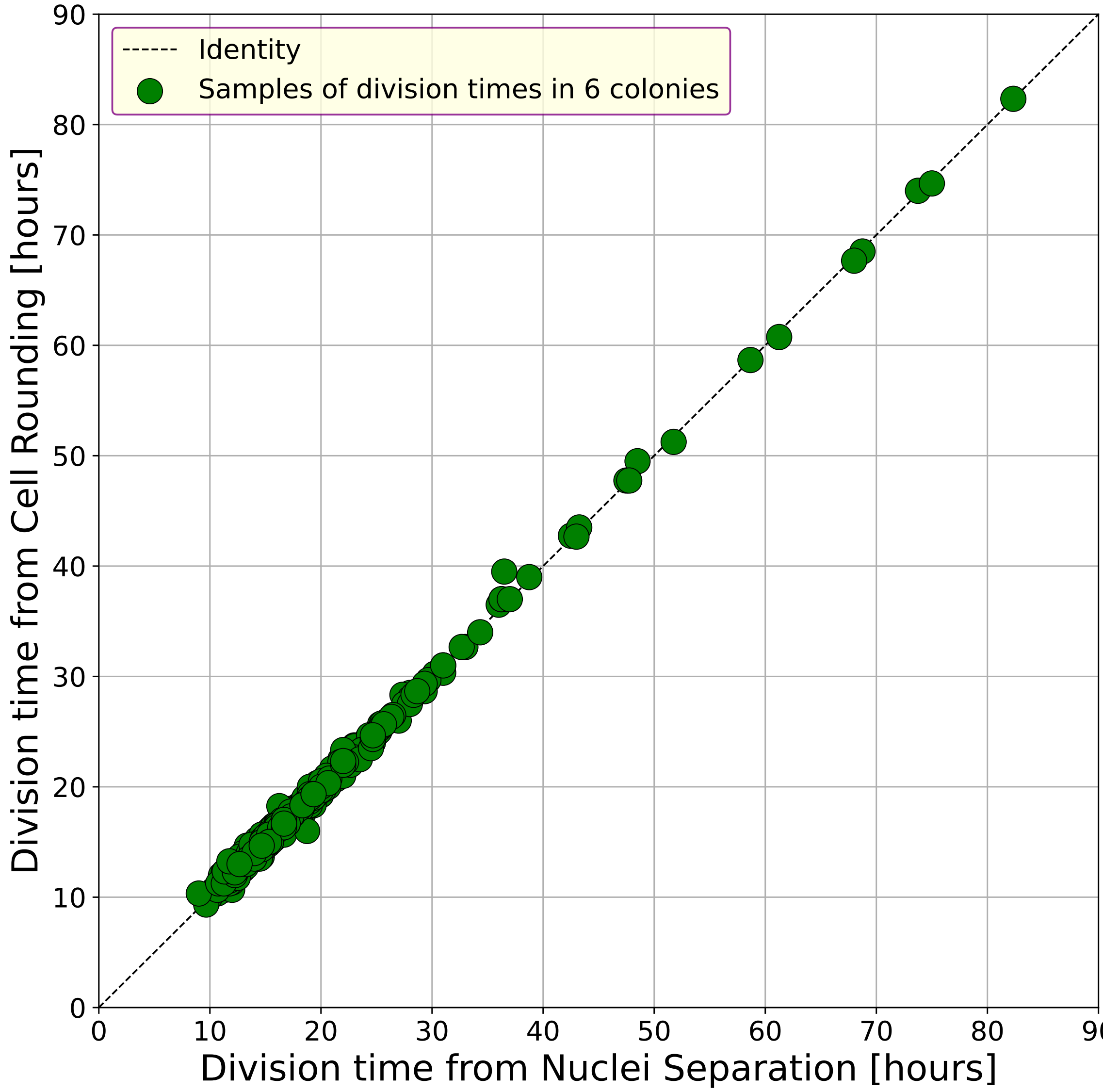

Figure S6: Different ways to determine the division times. We determine the division time, i.e. the duration of the cell cycle, through the mitotic cell rounding, a very short phase during which the cell assumes an almost spherical shape, appearing as a bright, circular object. This trait is easily recognisable in the image, which is why we use it an unambiguous time stamp of the cell cycle. An alternative time stamp of mitosis is the first image at which the two daughters' nuclei are clearly separated from each other; this criterion, however, has a larger degree of arbitrariness and it is operator-dependent, because the exact time at which the two nuclei become clearly separated depends on the morphology of those particular cells. In this figure, we plot against each other these two possible determinations of the division time, for the same 6 colonies as in Figs.2, 3, 6 of the main text. The perfect correlation shows that the two definitions are completely equivalent.

References

1. Deena A Rennerfeldt, Joana S Raminhos, Samantha M Leff, Pristinavae Manning, and Krystyn J Van Vliet. Emergent heterogeneity in putative mesenchymal stem cell colonies: Single-cell time lapsed analysis. *PLoS One*, 14(4):e0213452, 2019.
2. Maria Surovtseva, Nikolay Surovtsev, Natalia A Bondarenko, Irina Kim, Alexander Lykov, and Olga Poveshchenko. Survival and proliferation of bone marrow derived mesenchymal stem cells under 532 nm laser irradiation. In *2021 IEEE Ural-Siberian Conference on Computational Technologies in Cognitive Science, Genomics and Biomedicine (CSGB)*, pages 160–163. IEEE, 2021.
3. P Philippe Laissue, Rana A Alghamdi, Pavel Tomancak, Emmanuel G Reynaud, and Hari Shroff. Assessing phototoxicity in live fluorescence imaging. *Nature methods*, 14(7):657–661, 2017.
4. Wei-Kee Ong, How-Foo Chen, Cheng-Ting Tsai, Yun-Ju Fu, Yi-Shan Wong, Da-Jen Yen, Tzu-Hao Chang, Hsien-Da Huang, Oscar Kuang-Sheng Lee, Shu Chien, et al. The activation of directional stem cell motility by green light-emitting diode irradiation. *Biomaterials*, 34(8):1911–1920, 2013.
5. Ye Yuan, Gege Yan, Rui Gong, Lai Zhang, Tianyi Liu, Chao Feng, Weijie Du, Ying Wang, Fan Yang, Yuan Li, et al. Effects of blue light emitting diode irradiation on the proliferation, apoptosis and differentiation of bone marrow-derived mesenchymal stem cells. *Cellular Physiology and Biochemistry*, 43(1):237–246, 2017.
6. Yuguang Wang, Ying-Ying Huang, Yong Wang, Peijun Lyu, and Michael R Hamblin. Red (660 nm) or near-infrared (810 nm) photobiomodulation stimulates, while blue (415 nm), green (540 nm) light inhibits proliferation in human adipose-derived stem cells. *Scientific reports*, 7(1):7781, 2017.
7. Hao Li, Shifen Wang, Yining Hui, Yajiao Ren, Jiaxin Li, Xiaorong Lan, and Yao Wang. The implication of blue light-emitting diode on mesenchymal stem cells: a systematic review. *Lasers in Medical Science*, 38(1):267, 2023.
8. Giada Magni, Martina Banchelli, Leonardo Ciaccheri, Marina Mazzoni, Barbara Adinolfi, Lucia Cavigli, Roberto Pini, and Francesca Rossi. Can photobiomodulation be studied in vitro? investigation on the interaction between cell culture medium and short-wavelength blue led light. In *Optical Interactions with Tissue and Cells XXXV*, volume 12840, pages 92–96. SPIE, 2024.
9. Gary Bradski. The opencv library. *Dr. Dobb's Journal: Software Tools for the Professional Programmer*, 25(11):120–123, 2000.
10. Anna V Taubenberger, Buzz Baum, and Helen K Matthews. The mechanics of mitotic cell rounding. *Frontiers in cell and developmental biology*, 8:687, 2020.
11. Manuel Thery and Michel Bornens. Get round and stiff for mitosis. *HFSP Journal*, 2(2):65–71, 2008.
12. Theodore Edward Harris. *The theory of branching processes*, volume 6. Springer Berlin, 1963.
13. N. G. van Kampen. *Stochastic Processes in Physics and Chemistry*. Elsevier, Amsterdam, 3 edition, 2007.
14. Natalia G Lavalle, Osvaldo Chara, and Tomás S Grigera. Fluctuations in tissue growth portray homeostasis as a critical state and long-time non-markovian cell proliferation as markovian. *Royal Society Open Science*, 10(9):230871, 2023.
15. Bruna Pucci, Margaret Kasten, and Antonio Giordano. Cell Cycle and Apoptosis. *Neoplasia*, 2(4):291–299, July 2000.
16. Panteleimon Rompolas, Kailin R Mesa, Kyogo Kawaguchi, Sangbum Park, David Gonzalez, Samara Brown, Jonathan Boucher, Allon M Klein, and Valentina Greco. Spatiotemporal coordination of stem cell commitment during epidermal homeostasis. *Science*, 352(6292):1471–1474, 2016.
17. R.G. Staudte, M. Guiguet, and M. Collyn d'Hooghe. Additive models for dependent cell populations. *Journal of Theoretical Biology*, 109(1):127–146, July 1984.
